# How are European small enterprises dealing with the circular economy?

**DOI:** 10.12688/openreseurope.23303.2

**Published:** 2026-10-06

**Authors:** Maria Pilar García-Alcober, Ana Isabel Mateos-Ansótegui, María Teresa Pastor-Gosálbez, Josep Navarro-Ortiz

**Affiliations:** 1 Universidad CEU Cardenal Herrera Departamento de Economia y Empresa, Alfara del Patriarca, Valencian Community, Spain; 2 Universitat de Valencia, Valencia, Valencian Community, Spain

**Keywords:** Circular Economy; Clusters; European SME

## Abstract

**Background:**

Environmental sustainability and energy savings are crucial for economic development, yet European SMEs account for 63.3% of total CO2 firms emissions. Understanding how these businesses behave when transitioning to a Circular Economy is essential for policymakers to design appropriate measures and incentives for preventing negative environmental externalities.

**Methods:**

The study analyzed data from the fifth wave of the “SMEs, resource efficiency, and green markets” survey conducted in late 2021, which included 14,008 enterprises across 27 European Union member states. To categorize the companies, researchers applied a K-modes clustering algorithm—a machine learning method designed specifically for categorical data— to evaluate six qualitative business characteristics and nine environmental actions.

**Results:**

The analysis identified three distinct business behavior clusters. Cluster 1 (6,195 firms) primarily provides services to other businesses and focuses heavily on energy savings and waste minimization. Cluster 2 (4,002 firms) is the least environmentally proactive group, predominantly selling products directly to consumers, with 21.14% taking no environmental action at all. Cluster 3 (3,811 firms) is the most active and diverse group, exhibiting the highest participation in minimizing waste and saving energy but also reporting the most difficulties. Regardless of the cluster, the primary barrier to sustainability is the complexity of administrative or legal procedures, followed by the high costs of environmental actions. Consequently, the support most requested by these companies consists of grants or subsidies along with greater cooperation between companies across sectors and consultancy.

**Conclusions:**

The transition to sustainable practices remains a significant challenge for most European SMEs, who often perceive current macro-level environmental regulations (such as the Circular Economy Action Plan) as bureaucratic hurdles rather than sources of help. To effectively foster a sustainable economy, policymakers must focus on simplifying administrative procedures, providing financial support, and encouraging inter-firm cooperation and technological consultancy.

## 1. Introduction

Climate change, resource scarcity, and rising energy prices drive economic actors, especially businesses, to focus on efficiency and sustainability. In response to these concerns, in 2015, the European Commission implemented its first action plan for a Circular Economy. This plan aimed to transform the traditional linear economic model into a circular model, promoting the recycling and reuse of products to generate environmental benefits, reduce negative externalities, and enhance business benefits. According to Geissdoerfer et al. (2017), the Circular Economy is a regenerative system in which resource input and waste, emissions and energy leakage are minimized through slowing, closing and narrowing material and energy loops. This broader perspective extends beyond recycling and includes strategies aimed at preserving the value of products, components and materials throughout their life cycle.

Economic growth in recent decades has been described as unsustainable ( Ghisellini et al., 2016), largely because it is based on a linear model. This type of model uses resources and raw materials discarded at the end of the production process, leading to a very negative environmental impact. By contrast, the Circular Economy model aims to replace the linear economy in production and consumption systems, replacing the ‘use and throw away’ approach with one that seeks to minimize waste as much as possible ( Christis et al., 2019; Turcu and Gillie, 2020).

The Circular Economy has become one of the main policy instruments through which the European Union seeks to contribute to the Sustainable Development Goals, particularly SDG 12 (Responsible Consumption and Production) and SDG 13 (Climate Action). Consequently, European SMEs play a pivotal role because they represent the largest share of businesses in Europe and are directly affected by the regulatory framework derived from the Circular Economy Action Plan.

However, achieving a Circular Economy requires a series of changes in production processes, either by increasing energy efficiency or reusing materials. The transition from a linear to a Circular Economy has become a political priority at both the international and local levels, and this has led to the enactment of legislation to drive firms to change their production processes.

The Circular Economy Action Plan initially focused on ecological and economic issues ( European Commission, 2015, 2020). Nevertheless, the European Union currently finds itself in a complex geopolitical landscape ( Baldassarre, 2025). As a result, some industries have been experiencing a scarcity of basic resources and problems with their energy supply in recent years. In response, the European Parliament reacted to The Critical Raw Materials Act ( European Parliament and Council, 2024).

The business sector is responding accordingly, both to comply with current legislation and to reduce costs, prompted by growing awareness of the need for environmental sustainability and to reduce their dependence on international suppliers. In this context, it is especially important to understand the firms’ current situation, to identify the initiatives they are implementing, the obstacles they face in the transition towards sustainable production, and, above all, whether environmental administrations and legislation act as facilitators or barriers to achieving sustainable economic growth.

In this study, we focus on small and medium-sized firms (SMEs) in countries that comprise EU-27. Although European SMEs have limited environmental impact at the individual level, they account for 63.3% of the total CO _2_ firms emissions in Europe, according to the European Commission’s annual report on European SMEs 2021/22 (2022b).

Understanding business behavior in this area and the difficulties and challenges they face is essential to implement appropriate policies to incentivize the reduction of these negative externalities.

Studies on SMEs and sustainability, such as Chatzistamoulou and Tyllianakis (2022), identify factors that influence firms to implement and commit to renewable efficiency actions. A similar approach was adopted by Vasilescu et al. (2023), although their study focused on entrepreneurship and the supply of green products and services.

Unlike studies that analyze the factors that influence firms’ decisions to adopt more environmentally sustainable policies, this study focuses on the current situation. As such, it has a twofold objective: on the one hand, to analyze the responses of firms by country to the environmental measures proposed in the EU regulation, and on the other hand, to categorize firms in terms of the actions taken in response to these environmental measures on a country-by-country basis.

According to the Circular Economy hierarchy, circular strategies differ substantially in their capacity to preserve the value of products, components and materials. Actions aimed at extending product life, promoting reuse, repair or industrial symbiosis generally represent higher levels of circularity than resource-efficiency measures such as saving water or energy. Consequently, the environmental practices analysed in this study should not be interpreted as equivalent but rather as complementary dimensions of firms’ transition towards Circular Economy principles. The remainder of this paper is organized as follows. In Section 2, we examine the methodology used; this, in turn, is divided into three parts. First, we set out the database used and the descriptions of the sample, and then we analyzed the clustering methodology used to categorize the firms. In Section 3, we consider the results, and in Section 4, we present the discussion and conclusions.

## 2. Methodology

In this study, we used data from the latest wave of the ‘SMEs, resource efficiency, and green markets’ survey, selecting European Union member states. This data is published as Flash Eurobarometer 498. SMEs, Resource Efficiency and Green Markets, wave 5 (2022a) First, we provide a descriptive overview of the survey data. We then identified the variables to be analyzed before performing a K-mode clustering analysis to categorize the companies.

### 2.1 Database

**2.1.1 Firm characteristics**

The database includes a set of variables that describe the structural characteristics of the firms surveyed. These variables capture differences in firms’ structural and market profiles, including country, sector of activity, firm size, recent turnover performance, type of output, and customer orientation. These characteristics are commonly associated with firms’ resources, capabilities, and market positioning, which may influence both their capacity and their incentives to adopt Circular Economy practices. The variables included in the analysis are presented in Table 1.

**Table 1.** Structural and market characteristics of the surveyed firms.

Variable	N° options	Options
Country	27 States	
Sector	12 sectores	(B/C/D/E/F/G/H/I/J/K/L/M)
Size	4	*Large* *Medium* *Small* *Micro*
Annual turnover growth	5	*Decreased* *Increased* *Remained unchanged* *Not applicable* *Don’t know, no answer*
Type of output	3	*Sell products,* *Sell services,* *Sell both*
Type of customer	8	All to the public administration; All to other companies; To other companies and the public administration; Directly to consumers; Directly to consumers and the public administration; Directly to consumers and other companies; Directly to consumers; Other companies and the public administration

Source: Own elaboration.

**Table 2.** Circular Economy practices included in the analysis and their corresponding circularity dimensions.

Circular Economy practice	Circularity dimension
Saving water	Reduce
Saving energy	
Saving materials	
Switching to greener suppliers of materials	
Minimising waste	
Using renewable energy	
Designing products that are easier to maintain, repair or reuse	Reuse
Selling your residues and waste to another company	
Recycling, by reusing material or waste within the company	Recycling

Source: Own elaboration.

**2.1.2 Circular economy practices**

The Eurobarometer survey includes a set of environmental practices capturing different dimensions of Circular Economy implementation. Some actions mainly focus on improving resource efficiency (e.g., saving water or energy), whereas others are more closely aligned with core Circular Economy strategies such as reuse, repair, recycling or industrial symbiosis. Consequently, the practices analyzed in this study should not be interpreted as having the same environmental relevance but rather as complementary dimensions of firms' transition towards Circular Economy principles. Following the Circular Economy literature, these practices can be grouped according to the main strategy they represent, ranging from resource-efficiency measures to actions aimed at closing material loops and extending product life. Each practice was coded as a binary variable indicating whether the firm reported implementing the corresponding action.

For ease of interpretation, these practices can be broadly grouped into four Circular Economy strategies. This categorization is descriptive only and is not used in the clustering procedure, which relies on the original binary variables.

Taken together, these variables provide a multidimensional representation of firms' engagement with Circular Economy practices and constitute the basis for the clustering analysis presented in Section 2.3.

### 2.2 Analysis of the business structure in Europe

We used the ‘SMEs, resource efficiency, and green market surveys as the basis for our analysis of European SMEs. The data used were from the latest wave of the survey, the fifth wave, which covers November and December 2021. We selected from the database only the 27 European Union member states. After the database was cleaned, (delating all missing values), there are a total of 14,008 firms in the survey sample. Below, we can see the main characteristics of the database cleaned: number of firms by country, age of the firms, main productive sector by country, distribution of the size of the firms by country and, finally, the type of client they target. Table 3 shows the distribution of the number of firms in the database by country.

**Table 3.** Number of firms per country that have answered the survey.

Country	No. Firms	Country	No. Firms	Country	No. Firms	Country	No. Firms
Austria	509 (3,6)	Finland	498 (3,6)	Latvia	503 (3,6)	R. of Cyprus	252 (1,8)
Belgium	587 (4,2)	France	599 (4,3)	Lithuania	499 (3,6)	Romania	609 (4,3)
Bulgaria	514 (3,7)	Germany	560 (4)	Luxembourg	248 (1,8)	Slovakia	496 (3,5)
Croatia	546 (3,9)	Greece	601 (4,3)	Malta	255 (1,8)	Slovenia	554 (4)
Czechia	596 (4,3)	Hungary	529 (3,8)	Netherlands	602 (4,3)	Spain	597 (4,3)
Denmark	492 (3,5)	Ireland	472 (3,4)	Poland	607 (4,3)	Sweden	593 (4,2)
Estonia	505 (3,6)	Italy	599 (4,3)	Portugal	586 (4,2)		

With regard to the age of the firms, we can observe that in 52% of the EU countries, the firms established after 2010 (considered the youngest) constitute the majority of respondents to the survey. The longest-established firms represented only 4% of the base, as shown in Table 4.

**Table 4.** Percentage of firms in the base by date of creation.

Established before 1970	Established between 1970 and 1990	Established between 1990 and 2010	Established after 2010
4%	15%	30%	52%

Source: Own elaboration.

The survey allowed us to distinguish between the different production sectors. We can see that the predominant sector is G (wholesale and retail trade and repair of motor vehicles and motorbikes) across all countries except Sweden and Estonia, where C (manufacturing) predominates.

Table 5 shows the percentages for each productive sector.

**Table 5.** Productive sector by percentage.

PRODUCTIVE SECTOR	%
B - Mining and quarrying	1.05
C - Manufacturing	20.21
D - Electricity, gas, steam and air conditioning supply	1.86
E - Water supply, sewerage, waste management and remediation activities	1.51
F - Construction	17.25
G - Wholesale and retail trade, repair of motor vehicles and motorcycles	28.16
H - Transportation and storage	7.25
I - Accommodation and food service activities	6.49
J - Information and communication	5.14
K - Financial and insurance activities	3.17
L - Real estate activities	2.38
M - Professional, scientific and technical activities	5.54

Source: Own elaboration.

We analyzed the invoicing of firms in the survey from a triple perspective. First, we looked at the sums invoiced; second, we looked at the recipients – that is, who is being invoiced – and, third, at what is being invoiced (whether it is a service, a production, or both).

Regarding the sums invoiced, we observe in Table 6 that in countries such as Sweden, Denmark, and Belgium, there is a significant presence of firms invoicing more than 50 million.

**Table 6.** Distribution of firms by country with a turnover of over 50 million euros.

Country	No. Firms	% of firms < 2 m € (1840 firms)	% overall on each country
Greece	424	5%	71%
Latvia	406	5%	81%
Malta	188	2%	74%
**Country**	**No. Firms**	**% over those >50 m € (433 firms)**	**% overall in each country**
Belgium	54	12	9.2
Denmark	55	13	11.2
Sweden	55	13	9.3
***TOTAL***	***164***	***38***	

In contrast, Table 7 shows the countries with the smallest firms, with an average turnover of less than 2 million.

**Table 7.** Distribution of firms by country with turnover below 2 million euros.

Country	No. Firms	% of firms < 2 m € (1840 firms)	% overall in each country
Greece	424	5	71
Latvia	406	5	81
Malta	188	2	74
Romania	461	6	76
Slovakia	361	4	73
***TOTAL***	***1840***	***23***	

The geographical concentration of the firms with the highest turnover is noteworthy. The 164 firms with a turnover of more than €50 million were located in Sweden, Denmark, and Belgium, although only 1% (164/14008) of the entire base of firms surveyed accounted for 38% (164/433) of all firms with a turnover above this amount. For example, in Sweden, 9.3% of all firms surveyed reported a turnover of more than €50 million, constituting 13% of all European firms with a turnover of more than €50 million.

Regarding the firms reporting the lowest turnover (below €2 million), those in Greece, Latvia, Malta, Romania, and Slovakia accounted for 13% (1840/14008) of the surveyed firm base, as shown in Table 5, while they accounted for 23% (1840/8069) of all firms with a turnover below this amount. For example, in Latvia, 81% of the surveyed firms had a turnover below EUR 2 million, but only 5% of all European firms had a turnover below this threshold.

For the typology of customers, we studied firms according to their main client base. The questionnaire answer options were as follows: all to the public administration; all to other firms; and the public administration; directly to consumers; directly to consumers and the public administration; directly to consumers and other firms; directly to consumers; and to other firms and the public administration.

We find that, across all countries, a higher percentage of firms do not have the government as a customer. However, this distribution was not symmetrical. Figure 1 shows that in Finland and Sweden, the percentage of firms that have public administration as a customer is as high as 40%, while in Portugal and Bulgaria, this percentage is less than 20%.

**Figure 1.**
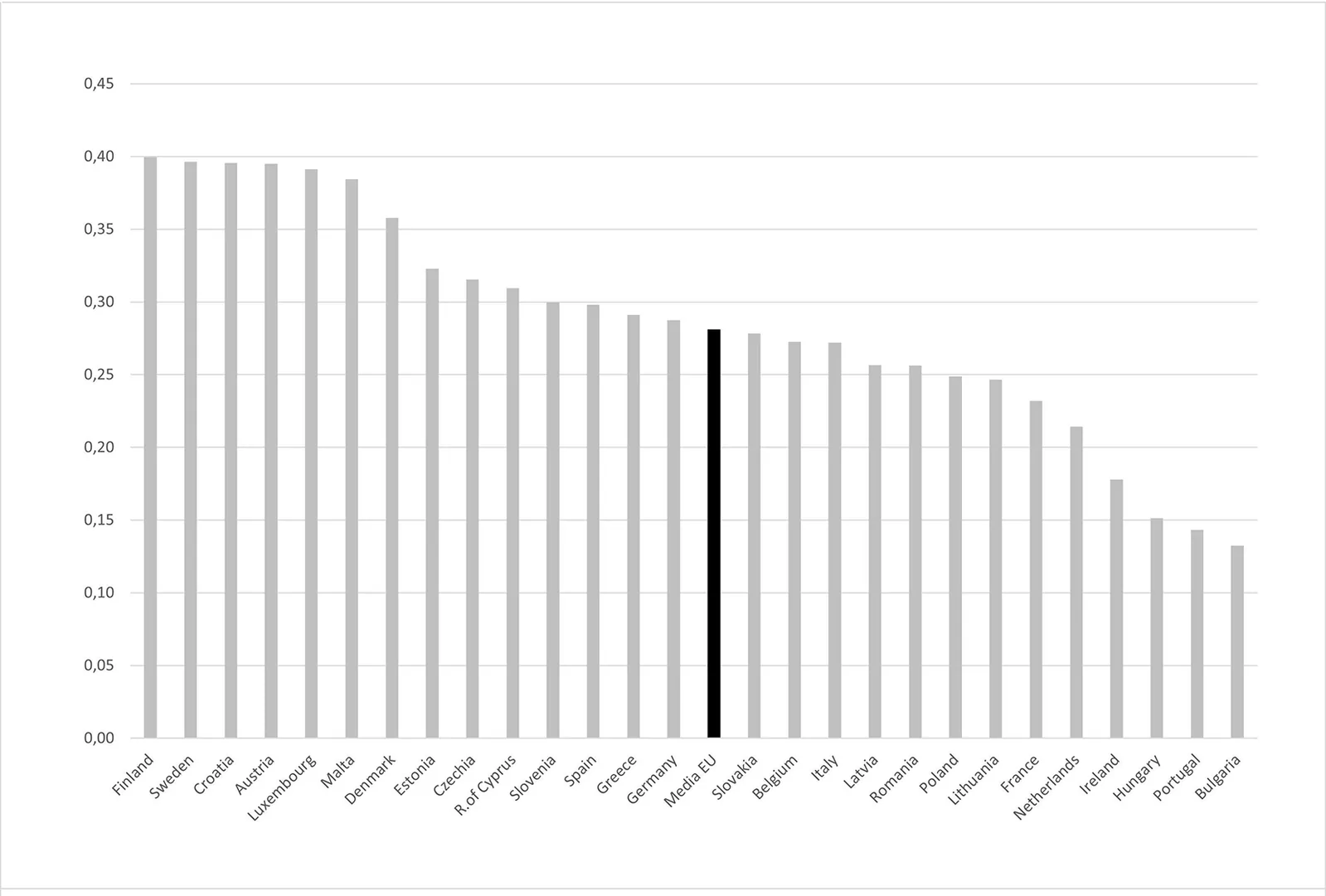
Percentage of firms by country contracting with public administration. Source: Own elaboration.

Finally, in terms of invoicing, Table 8 shows an even distribution between firms that sell only products, only services, or both.

**Table 8.** Number of firms that sell products, services or both.

OUTPUT	No. Firms	%
Both products and services	4669	33.33%
Products	4416	31.52%
Services	4923	35.14%
TOTAL	14008	100%

Source: Own elaboration.

The distribution across the firms is shown in Figure 2. For each country, the distribution of firms according to whether they sell products, services, or both, is shown as a percentage. Thus, Croatia and Finland firms that sell both services and products dominate. Italy and Portugal show a higher percentage of firms selling services only, while Malta and Cyprus stand out for selling products.

**Figure 2.**
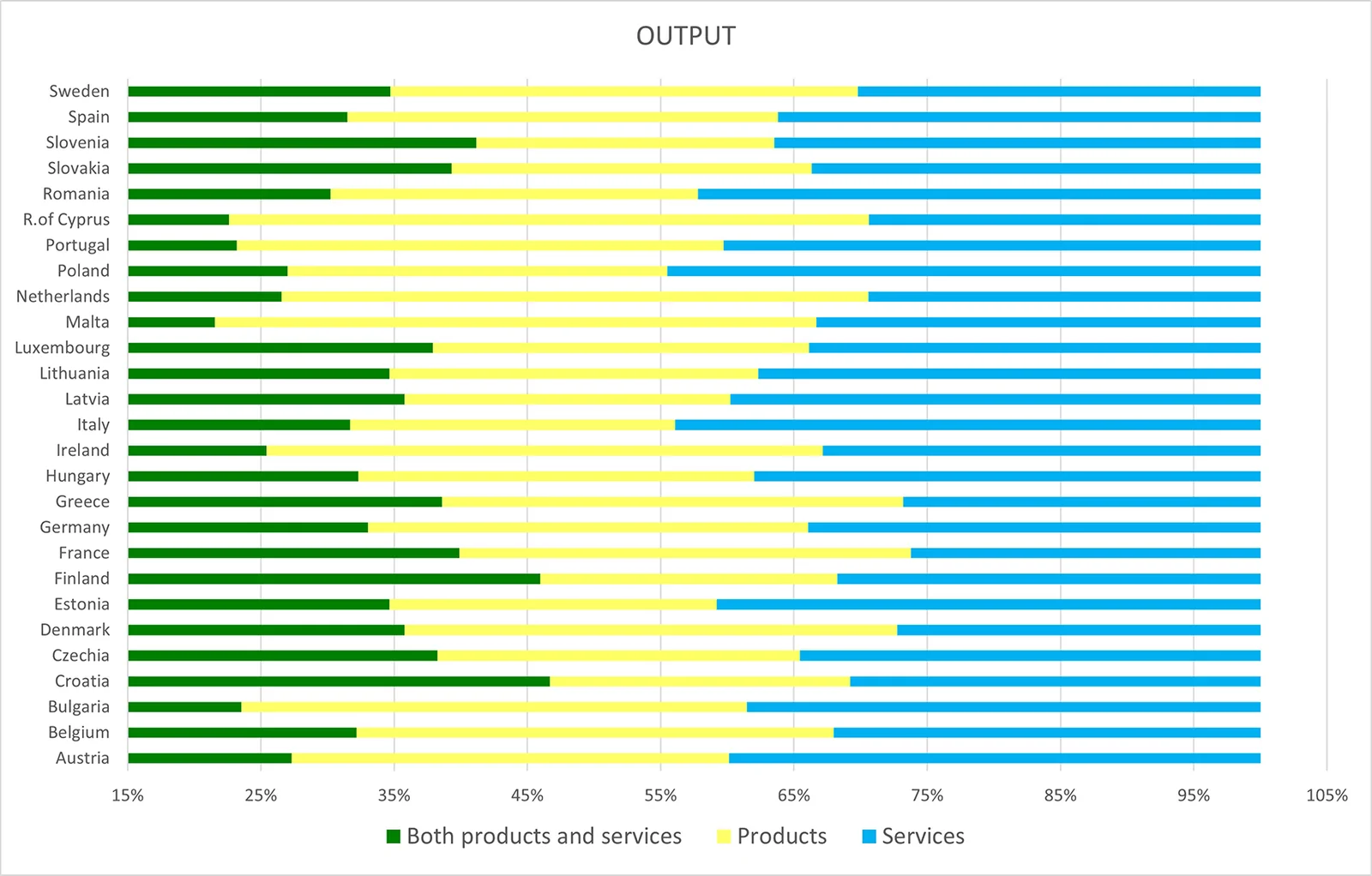
Distribution of the type of output sold by firms by country. Source: Own elaboration.

### 2.3 Method

In the second stage, a clustering analysis is performed based on the distribution of the firms’ responses in each of the countries included in the database. This study identifies groups of firms according to their environmental policies and business characteristics using categorical variables. For this, we use the K-modes method, ^1^ although in a different way to studies such as Gomonov et al. (2021), which use the K-means method. The K-mode clustering algorithm ( Cao et al., 2009), an adaptation of the K-means algorithm, was specifically designed for clustering categorical data. The K-mode clustering method is an unsupervised machine learning technique used to group a set of data objects into a specific number of groups based on qualitative variables. The algorithm is called ‘K-modes’ because it uses modes (i.e., the most frequent values) instead of means or medians to represent the groups. (The development and explanation of the proposed K-modes model are presented in Appendix I.).

The qualitative business variables that we take into account for the K-modes analysis are six variables that characterize the firms (country and productive sector in which they operate, size, level of growth, type of product, and type of customer) and nine variables that reflect the environmental actions implemented by the 14008 firms in the database (all the variables are described in section 2.1).

In the third stage, we analyze the problems and requirements of firms to become more environmentally sustainable. This information is valuable for the optimal design of environmental policies. As such, we use the answers given by the firms in the survey to the following questions: “Did your company encounter any of the following difficulties when trying to set up resource efficiency actions?” and “Which of the following will help your company the most to be more resource efficient?”

## 3. Results

This section presents the results of this study. First, the analysis of the environmental actions carried out by the firms in the sample, taking into account the country in which they operate, allows us to identify in which country each action predominates. Second, we analyze the clustering results by identifying groups of firms and their characteristics. Finally, we detect the difficulties experienced by firms and support that they need to be sustainable and identify the differences between the clusters.

### 3.1 Environmental actions carried out by firms in each country

We then analyzed the actions that firms take to make their resources more efficient and thus avoid negative externalities in the environment. The actions are classified according to the following list: saving water (SW); designing products that are easier to maintain, repair, or reuse (DP); recycling, reusing materials or waste within their company (R); selling their waste and residues to another company (SR); minimizing waste (MW); switching to greener material suppliers (GR); saving energy (SE); saving materials (SM); using predominantly renewable energy (including own production with solar panels, etc.) (RE); other; or none.

Table 9 shows the number of actions developed by firms according to country. Thus, we can see which type of activity each country focuses more on. It can be seen that, in general, waste minimization MW) and/or energy saving (SE) actions predominate in all countries. In general, countries show homogeneous behavior. There are some exceptions, such as Ireland and Portugal, where recycling is prioritized and Malta, where material and water savings are prioritized. If we look at Spain, its focus is to minimize waste and save materials, but what is most striking is that it is the country where water conservation has the highest priority. Of the Spanish firms, 72.19% saved water, a higher percentage than in other countries for this action.

**Table 9.** Percentage of a given action across all firms in a country.

	SW	DP	R	SR	MW	GR	SE	SM	RE	Others	None
Austria	44.99%	23.18%	47.94%	29.67%	65.62%	29.86%	66.21%	54.81%	31.43%	0.39%	
Belgium	55.03%	32.71%	46.51%	46.00%	83.48%	45.49%	76.32%	65.93%	45.66%	3.07%	
Bulgaria	25.10%	9.92%	24.51%	28.60%	39.88%	13.81%	42.02%	30.93%	9.14%	5.06%	
Croatia	44.87%	22.71%	35.90%	38.28%	68.13%	26.37%	56.59%	52.38%	10.07%	0.18%	
Czechia	47.99%	35.91%	47.48%	39.43%	74.50%	33.56%	64.60%	57.21%	13.59%	0.67%	
Denmark	29.88%	23.98%	31.10%	25.41%	43.29%	28.66%	54.67%	38.01%	22.97%	5.08%	
Estonia	32.28%	20.99%	34.26%	30.30%	51.49%	24.55%	51.68%	51.88%	12.67%	0.40%	
Finland	36.14%	31.33%	47.19%	31.73%	69.08%	35.74%	65.86%	65.26%	26.91%	2.41%	
France	40.07%	27.38%	47.75%	30.88%	55.43%	28.05%	47.25%	41.24%	10.18%	1.50%	
Germany	41.25%	27.50%	48.75%	33.04%	58.21%	38.04%	71.07%	53.39%	37.86%	1.07%	
Greece	39.77%	28.79%	45.26%	33.78%	52.58%	42.10%	56.41%	55.91%	15.97%	0.50%	
Hungary	55.77%	29.68%	36.11%	35.16%	66.16%	31.00%	73.91%	64.27%	23.25%	0.95%	
Ireland	28.18%	15.25%	53.60%	11.44%	50.85%	30.93%	40.47%	25.64%	12.71%	1.91%	
Italy	42.40%	23.21%	45.08%	25.21%	82.97%	32.89%	61.94%	59.43%	26.21%	1.17%	
Latvia	38.17%	23.66%	24.25%	28.63%	46.52%	27.63%	57.65%	55.27%	5.77%	2.39%	
Lithuania	53.51%	25.45%	25.25%	37.47%	59.32%	37.68%	65.33%	64.93%	12.42%	2.00%	
Luxembourg	41.53%	35.48%	46.37%	29.03%	67.34%	43.95%	55.65%	50.00%	14.52%	4.84%	
Malta	49.02%	21.18%	43.53%	23.92%	49.80%	38.43%	41.57%	49.41%	27.84%	3.92%	
Netherlands	38.70%	33.72%	56.64%	40.86%	76.08%	40.70%	75.75%	63.46%	50.33%	0.66%	
Poland	50.25%	17.63%	31.30%	30.97%	57.50%	27.35%	64.25%	60.63%	14.66%	1.15%	
Portugal	37.20%	15.87%	53.92%	25.43%	32.76%	19.97%	50.68%	41.64%	11.09%	4.44%	
R. of Cyprus	35.71%	13.10%	43.65%	19.84%	37.70%	25.40%	58.33%	46.43%	21.03%	0.00%	
Romania	63.88%	35.80%	44.17%	45.32%	76.03%	45.98%	70.61%	73.07%	11.00%	0.82%	
Slovakia	70.77%	31.45%	52.82%	30.04%	79.03%	40.52%	74.80%	73.79%	9.07%	0.40%	
Slovenia	36.28%	23.29%	39.89%	38.27%	63.72%	38.81%	54.87%	47.83%	17.51%	2.35%	
Spain	72.19%	39.20%	76.72%	45.06%	85.43%	52.60%	83.75%	84.09%	24.46%	1.01%	
Sweden	46.21%	47.89%	70.99%	40.47%	85.67%	60.03%	77.91%	82.80%	48.57%	1.35%	

Source: Own elaboration.

Next, we focused on the overall set of shares. Table 10 shows the average number of actions taken by firms in each country. Spanish firms report the highest number of actions on average (an average of 5.64 actions per firm out of nine possible 9 actions), closely followed by Swedish firms (an average of 5.62 actions per company). At the other end of the scale, Bulgaria is the country where firms report the lowest average number of actions (2.29 actions per firm).

**Table 10.** Average number of actions by firms in each country.

Country	Firms per Country	Number of total actions	Number of total actions in average	Country	Firms per Country	Number of total actions	Number of total actions in average
Austria	509	2006	3.94	Latvia	503	1559	3.10
Belgium	587	2936	5.00	Lithuania	499	1913	3.83
Bulgaria	514	1177	2.29	Luxembourg	248	964	3.89
Croatia	546	1941	3.55	Malta	255	889	3.49
Czechia	596	2473	4.15	Netherlands	602	2871	4.77
Denmark	492	1491	3.03	Poland	607	2159	3.56
Estonia	505	1568	3.10	Portugal	586	1717	2.93
Finland	498	2050	4.12	R. of Cyprus	252	759	3.01
France	599	1975	3.30	Romania	609	2842	4.67
Germany	560	2297	4.10	Slovakia	496	2295	4.63
Greece	601	2230	3.71	Slovenia	554	2010	3.63
Hungary	529	2202	4.16	Spain	597	3370	5.64
Ireland	472	1279	2.71	Sweden	593	3332	5.62
Italy	599	2399	4.01				

Source: elaboration of our own.

We now group the nine actions into three figures ( Figures 3, 4, and 5) to analyze the performance of each country against each of the environmental actions studied.

**Figure 3.**
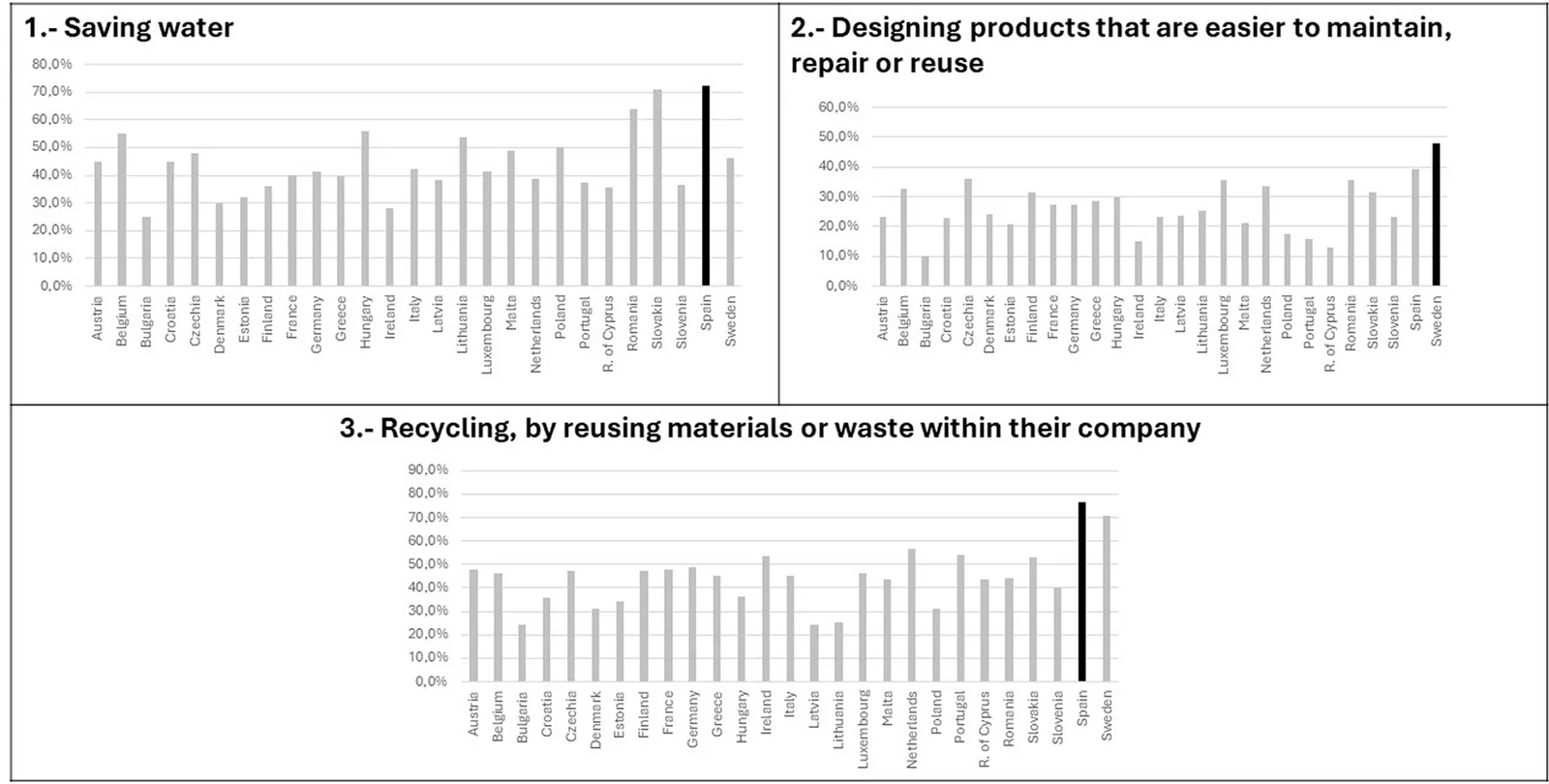
Actions: a. ‘Saving water’; b. ‘Designing products that are easier to maintain, repair or reuse’; c. ‘Recycling, by reusing materials or waste within their company’. Source: Own elaboration.

**Figure 4.**
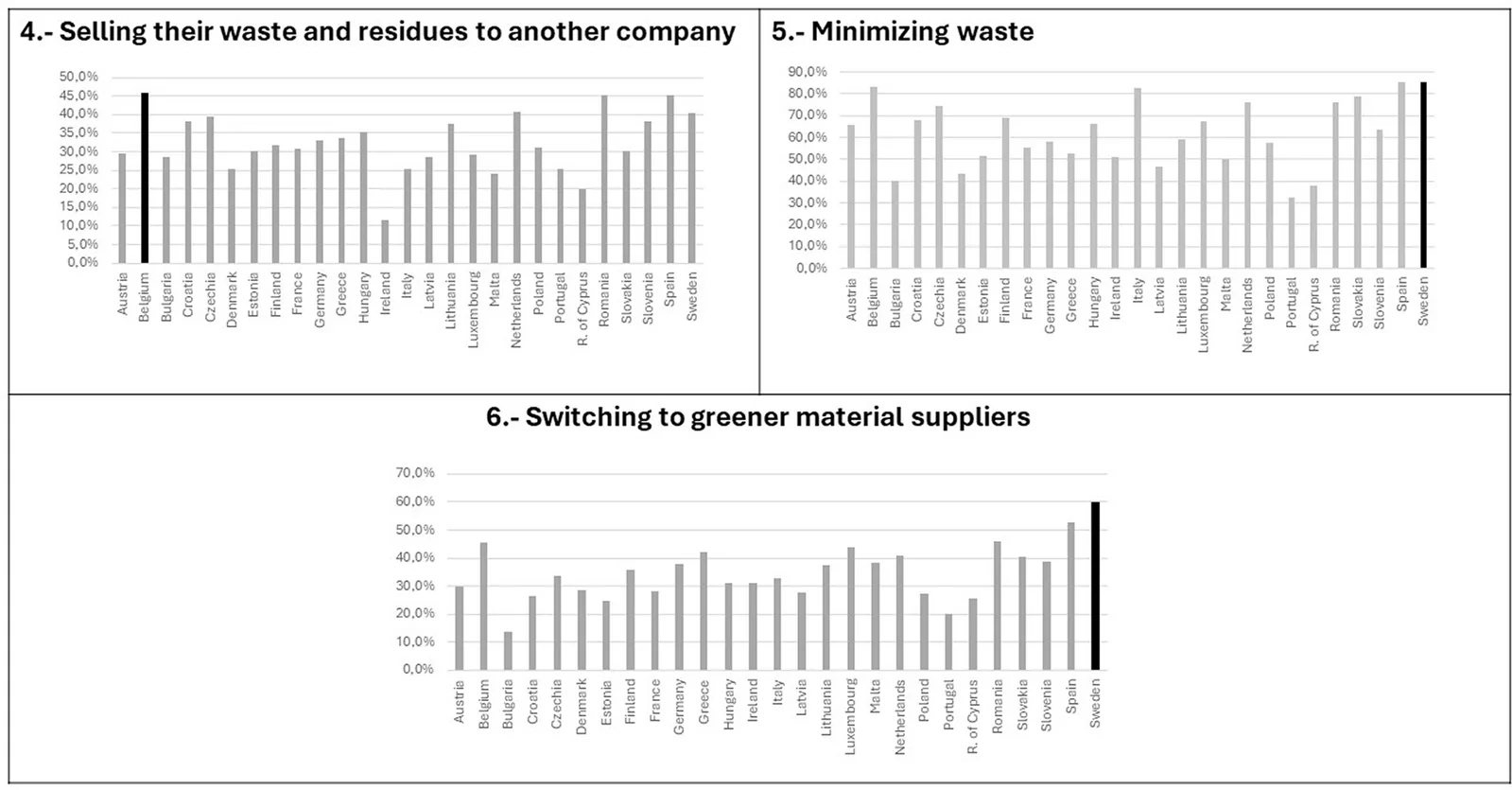
Actions: d. ‘Selling their waste and residues to another company’; e. ‘Minimizing waste’; f. ‘Switching to greener material suppliers’. Source: Own elaboration.

**Figure 5.**
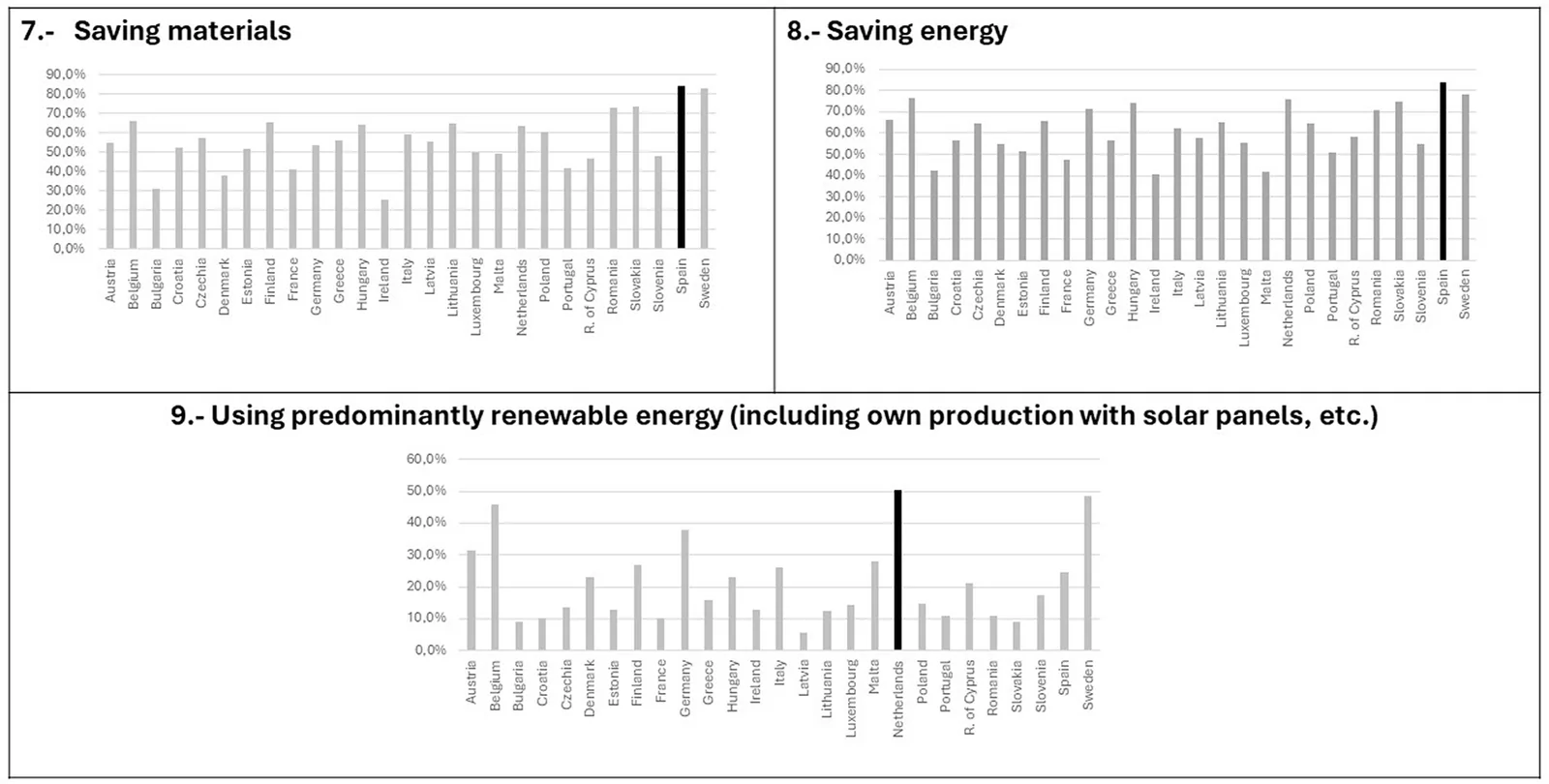
Actions: g. ‘Saving materials’, h. ‘Saving energy’ y i. ‘Using predominantly renewable energy (including own production with solar panels, etc.)’ Source: Own elaboration.

Regarding Action a (water saving), the results show that Spanish firms are the best performers, with 72.2%, closely followed by Slovakian firms. Bulgarian and Irish firms have the least importance for this action.

The objective of Action b ‘Design products that are easier to maintain, repair, or reuse is to move away from the attitude of the linear economy to avoid excessive waste that degrades the environment.’ However, this action is least directly related to economic savings. As can be seen, Swedish firms, with 47.9%, give most importance to this action.

Action c ‘Recycle, by using materials or waste within your company,’ also sees Spanish firms stand out. A total of 76.72% stated that they carried out this action, followed by Swedish firms in second place.

Figure 4 shows that Belgian firms stand out for Action d ‘Sell your waste and residues to other firms,’ with 46% of firms reporting that they sell their waste; Romanian firms occupy second place. Regarding Action e (minimize waste), Swedish firms again stood out at 85.7%. For Action f (switch suppliers of more environmentally friendly materials), Swedish firms again stand out at 60%.

For Action g (‘Save energy’) and Action h (‘Save materials’), Spanish firms were once again leading the way, showing 83.8% and 84.1%, respectively. For Action i ‘Use predominantly renewable energy (including own production with solar panels, etc.), Dutch firms stand out from the rest at 50.3%.

Taking into account the three types of actions for which Swedish firms stand out (designing products that are easier to maintain, reduce waste, and look for greener suppliers), we can deduce that firms in this country are more aware of environmental sustainability, as these actions do not in the first instance lead to savings in terms of cost, but can even reduce economic profitability.

On the other hand, Spanish firms give more importance to saving water, energy, materials, and to recycling. This suggests that they are more concerned with cost savings in order to increase their competitiveness.

### 3.2 Clustering analysis

Using the K-mode clustering method, we could differentiate the three clusters. Cluster 1 consists of 6,195 firms, Cluster 2 consists of 4,002 firms, and Cluster 3 consists of 3,811 firms. Before examining the relationship between the environmental actions carried out by the firms and the clusters, we performed a significance analysis before drawing up the contingency tables.

Table 11 summarizes the main characteristics of the three clusters. These are the number of firms contained in each cluster, the Circular Economy activities they carry out, whether they sell products and/or services, and the type of customers they target.

**Table 11.** Number of firms and characteristics per cluster.

Cluster	Firms	Characteristics
Cluster 1	6195	Saving energy (71.65%), Minimizing waste (72.90%) Selling services (43,04%). Sell to other firms
Cluster 2	4002	Saving energy (22.31%), Recycling (29,51%) None (21,14%) Selling products (45,18%). Selling directly to consumers
Cluster 3	3811	Saving energy (89.50%), Minimizing waste (91,13%) Selling services and products (51,01%). Selling to other companies, directly to consumers or to the public administration

Source: Own elaboration.

As shown in Table 11, Cluster 1, consisting of 6,195 firms, is the largest cluster. The activities carried out by these firms in terms of sustainability were mainly energy saving (71.65% of the firms stated that they saved energy) and waste minimization (72.90% of the firms). They focus on selling the services to other firms.

The second group comprises 4,002 firms and is characterized by the fact that it is the group that performs the fewest actions. If we look at this group, the most common action is recycling, which is only carried out by 29.51% of the firms, followed by energy-saving (22.31% of the firms). In addition, 21.14% of the firms declared that they did not carry out any of the activities related to energy and/or resource saving. This cluster is also characterized by the fact that firms sell products to end consumers.

The third group, Cluster 3, was the smallest, consisting of 3,811 firms. It places the greatest emphasis on minimizing waste (91.13% of the firms) and/or saving energy (89.50% of the firms). It is more heterogeneous in terms of what they sell and to whom, as more than half of the firms sell services and products and their clients can be other firms, end consumers, or public administrations. This is the cluster in which firms declare more environmental actions, in contrast to Cluster 2.

According to Masi et al. (2018), CE initiatives can be carried out at three levels: the micro-, meso-, and macro-levels. They find evidence that most CE actions are at the micro level, and our findings concur with this. Micro-level initiatives refer to the 3R Principles: Reduce, Reuse, and Recycle. Meso-level initiatives refer to an inter-firm level, which in this study corresponds to actions: ‘Sell your waste and residues to other firms’ and ‘Switch to suppliers of more environmentally friendly materials.’ Macro-level initiatives cannot be checked directly in this survey, as they correspond to the government level.

Table 12 is a contingency table showing the relationship between the environmental variables and clusters.

**Table 12.** Cross-tabulation of environmental actions and clusters.

Actions	Cluster 1	Cluster 2	Cluster 3	EST- Chi^2	Cramer’s V
Minimizing waste	72.90%	21.94%	91.13%		
Saving materials	66.49%	14.52%	85.46%		
Saving energy	71.65%	22.31%	89.50%		
Saving water	40.02%	16.44%	82.29%		
Switching to greener suppliers of materials	25.31%	15.24%	71.69%		
Selling your residues and waste to another company	22.11%	18.44%	66.83%		
Recycling, by reusing material or waste within the company	35.50%	29.51%	76.36%		
None	2.57%	21.14%	0.00%		
Designing products that are easier to maintain, repair or reuse	24.39%	12.69%	46.29%		
Using renewable energy	20.48%	10.79%	33.61%		

Table 12 shows the relative weights, that is, the percentage of firms in each cluster that perform each of the different actions. For example, in Cluster 1, 72.9% of firms carried out the action of minimizing waste. In general, we can see that the firms in Cluster 3 are the most environmentally active because the percentage of firms participating in each of the actions is higher than that of the firms in the other two clusters.

Since these environmental actions constitute the variables used to generate the K-modes partition, Table 12 should not be interpreted as an independent statistical validation of the clustering. Instead, it provides a descriptive profile of the environmental characteristics that define each cluster.

Finally, the previously analyzed clustering results can be mapped, showing which cluster predominates in each EU country.

The map in Figure 6 shows the importance of each cluster for each country. Thus, Area A is composed of countries whose firms belong mainly to Cluster 1. In area B, we find the countries whose firms belong mainly to Cluster 2, and in C, we find those in Cluster 3. In areas X, Y, and Z, no single predominant cluster was observed. In area X, Clusters 1 and 2 were predominant. In area Y, Clusters 1 and 3 were predominant. In area Z, Clusters 2 and 3 show similar representations. To determine whether a particular cluster predominates in a country, we first calculated the average number of companies belonging to each cluster. If a country exceeds that average, we consider that cluster to be predominant in that country. Thus, there are countries where only one cluster predominates, and others where two clusters predominate at the same time. To summarize, we represent countries by area in Table 13.

**Figure 6.**
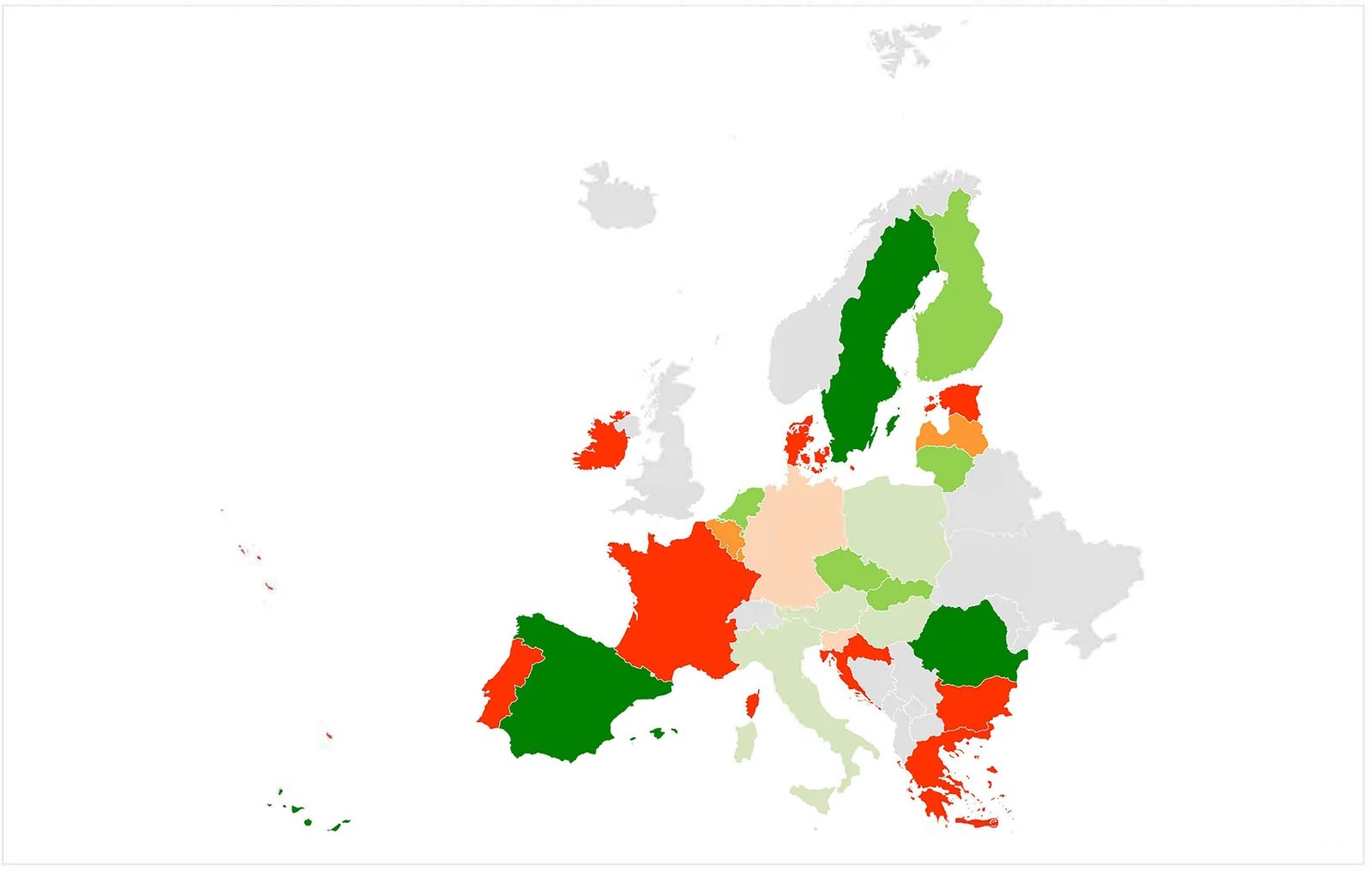
Predominant clusters in each country.

**Table 13.** Distribution of countries by area.

Area A	Area B	Area C	Area X	Area Y	Area Z
Austria	Bulgaria	Romania	Belgium	Czechia	Germany
Hungary	Croatia	Spain	Latvia	Finland	Slovenia
Italy	Denmark	Sweden	Luxembourg	Lithuania	
Poland	Estonia			Netherlands	
	France			Slovakia	
	Greece				
	Ireland				
	Malta				
	Portugal				
	R. of Cyprus				

Source: Own elaboration.

As can be seen, area C shows the countries that carry out the most actions, that is, where the firms that make up Cluster 3 predominate. However, this does not mean that they perform the same type of action. For example, as we have seen in Table 9 and as Vasilescu et al. (2023) have also found, Swedish firms focus more on the design of environmentally sustainable products, whereas Spanish firms focus more on cost savings such as saving water or recycling materials.

### 3.3 Barriers encountered by firms and type of support they request

In this section, we examine the firms’ perceptions of the barriers they face when implementing environmental policy measures. Additionally, we present the types of aid requested by these firms. Both variables are shown by the type of cluster to which firms belong.

Figure 7 shows how firms are most affected by the cost of environmental actions and administrative procedures. These results are in line with Rizos et al. (2016) and Garcés-Ayerbe et al. (2019) which conclude that financial constraints and administrative complexity are among the most frequently documented obstacles. Kirchherr et al. (2018) relativizes it, they consider regulatory barriers as main important, but not the among the five most pressing barriers. As might be expected, the firms that carried out the most actions, those in Cluster 3, reported the most difficulties.

**Figure 7.**
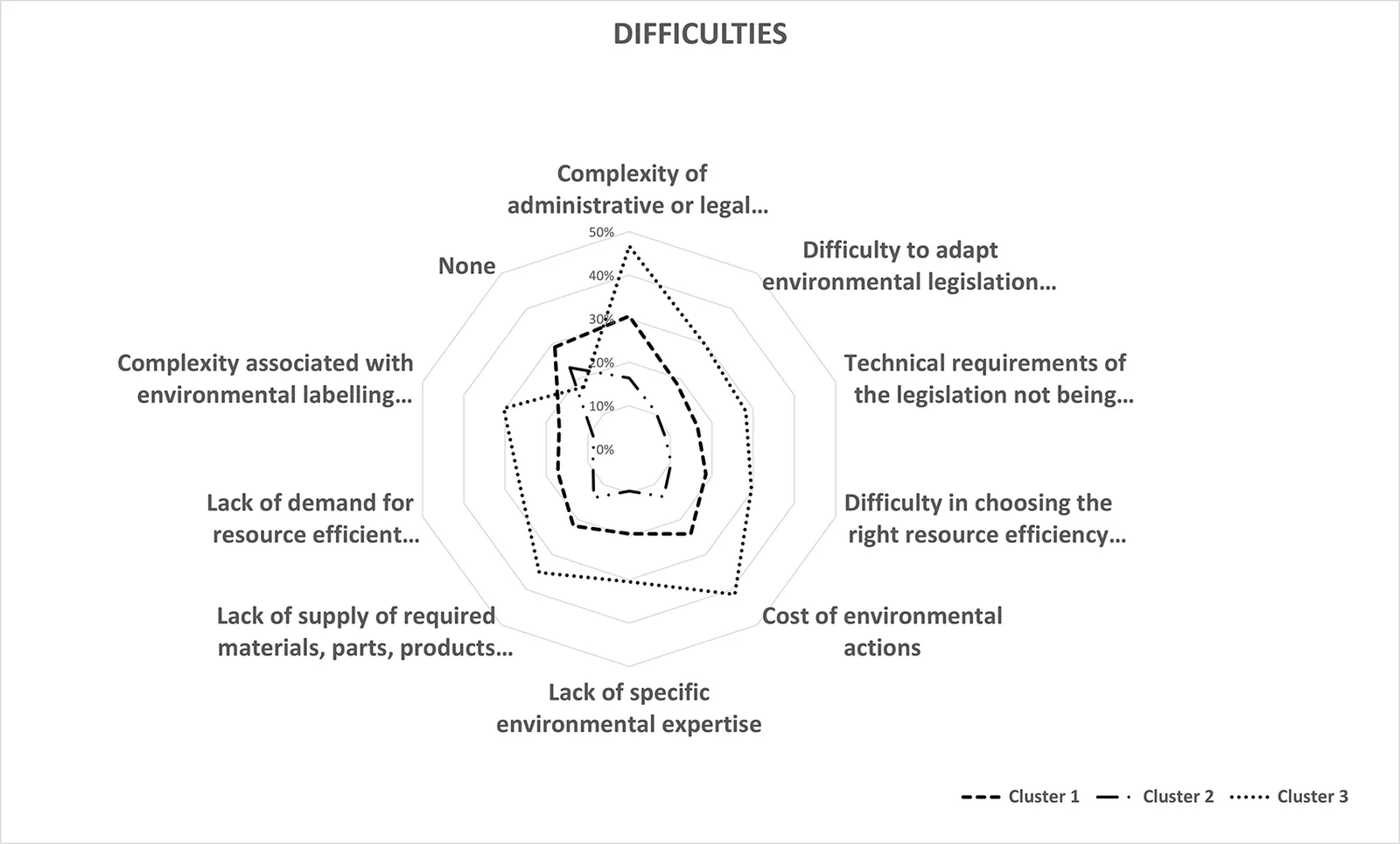
Distribution of clusters according to the perceived barriers faced by firms. Source: Own elaboration.

Table 14 presents a contingency analysis of these difficulties. This table confirms that administrative and legal procedures are perceived as the principal difficulties faced by firms in any cluster. This difficulty had the highest percentage of all clusters. That is, it does not depend on whether firms take more or fewer actions or on the type of action they take.

**Table 14.** Perceived barriers contingency analysis.

DIFFICULTIES	Cluster 1	Cluster 2	Cluster 3	EST- Chi^2	Cramer’s V
Complexity of administrative or legal procedures	30.64%	16.39%	46.71%	2826.97	0.449
The technical requirements of the legislation not being up to date	16.58%	8.80%	28.26%	2519.14	0.440
Difficulty in choosing the right resource efficiency actions for your company	18.60%	10.17%	29.57%	2510.67	0.440
Difficulty adapting environmental legislation to your company	18.64%	10.52%	29.70%	2494.65	0.435
Lack of specific environmental expertise	24.08%	13.54%	41.33%	2715.98	0.424
Lack of supply of required materials, parts, products or services	21.82%	13.79%	35.08%	2504.39	0.423
Lack of demand for resource efficient products or services	17.27%	8.72%	26.48%	2496.42	0.422
Complexity associated with environmental labelling and certification	17.27%	8.72%	26.48%	2496.42	0.422
Cost of environmental actions	24.08%	13.54%	41.33%	2715.98	0.440

Source: Own elaboration.

When analyzing the help sought by the firms, we can see in Figure 8 that they mainly request subsidies; however, in addition to this economic incentive to meet the cost of environmental actions, they also want greater cooperation with other firms. In terms of the significance of these variables, we can see that all are relevant.

**Figure 8.**
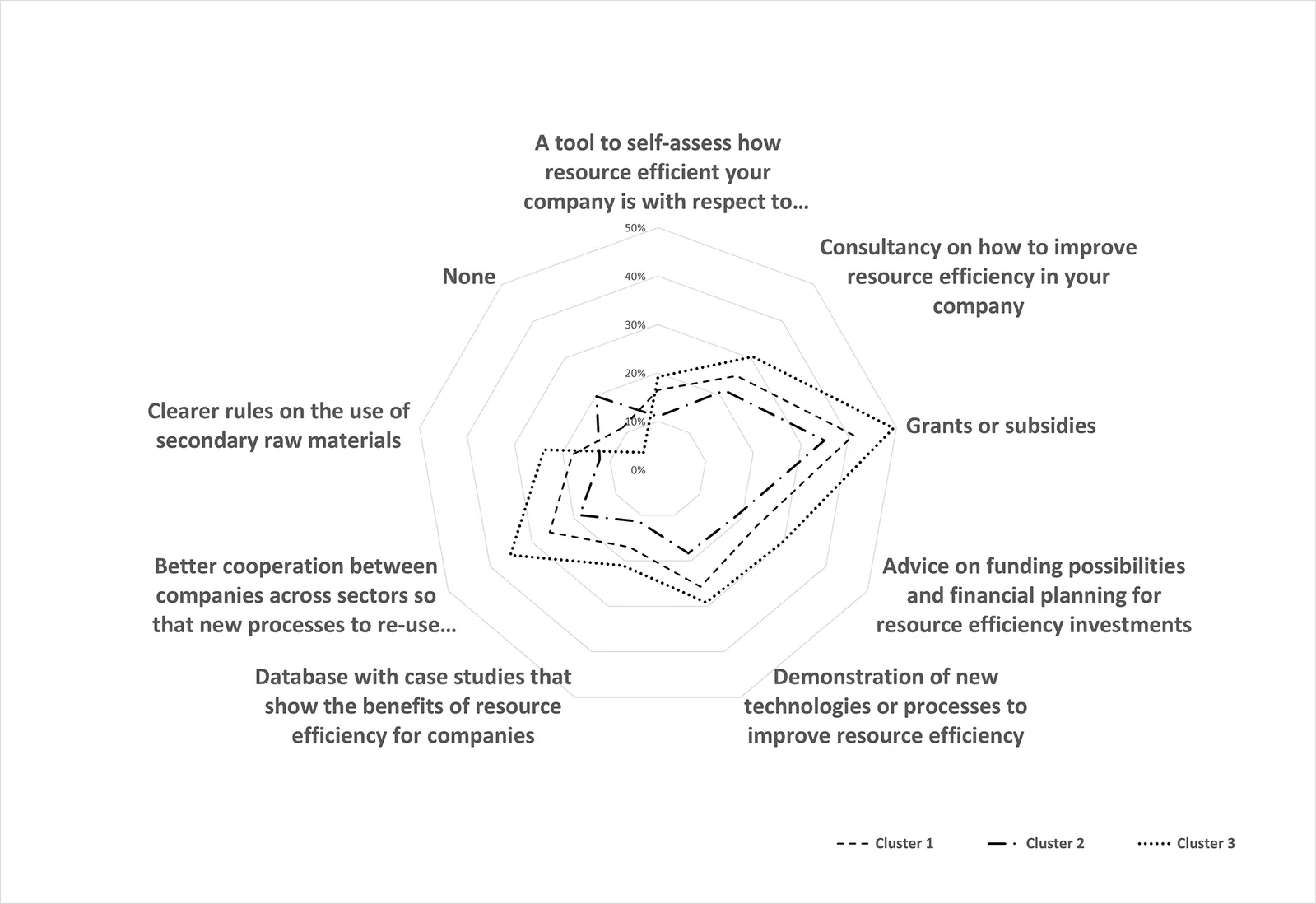
Distribution of clusters according to the support received by firms. Source: Own elaboration.

We can observe that the percentage of firms in Cluster 3, which are the most sustainable firms, are those that request more support. That is the reason we can see in Figure 8 “None” support is close to 0. There is also a relevant percentage of firms in the less environmentally active cluster that do not claim any aid.

A contingency analysis of these demands is presented in Table 15. All responses regarding the requested aid were significant. As shown in Figure 8, firms seek financial aid or subsidies as expected. However, it is striking that for firms in Clusters 1 and 3, the second most requested aid was cooperation between firms. However, for Cluster 2 (the least active in this area), the main request is for help in the form of consultancy on how to improve the efficiency of their use of resources.

**Table 15.** Requests contingency analysis.

Support requested	Cluster 1	Cluster 2	Cluster 3	EST- Chi^2	Cramer’s V
Better cooperation between companies across sectors so that new processes to reuse waste and byproducts can be …	25.83%	18.67%	35.16%	2388.75	0.415
A tool to self-assess how resource efficient your company is with respect to other companies	16.48%	10.97%	19.18%	2276.02	0.413
Clearer rules on the use of secondary raw materials	17.92%	12.19%	23.85%	2301.02	0.412
Advice on funding possibilities and financial planning for resource efficiency investments	23.42%	18.89%	29.76%	2270.74	0.405
Database with case studies that show the benefits of resource efficiency for companies	17.00%	11.37%	21.07%	2284.88	0.404
Demonstration of new technologies or processes to improve resource efficiency	25.71%	18.34%	29.18%	2373.70	0.403
Grants or subsidies	40.98%	34.91%	49.38%	2410.99	0.402
Consultancy on how to improve resource efficiency in your company	25.29%	21.39%	30.46%	2260.33	0.385

Source: Own elaboration.

## 4. Discussion

This study contributes to a better understanding of SMEs in the context of a Circular Economy in the EU. This is essential for policymakers, both for ecological reasons and given the uncertain geopolitical situation. We detected three clusters based on the company characteristics. Cluster 3 was the most proactive in terms of environmental issues and Cluster 2 was the least proactive. The following observations can be made. On the one hand, the EU’s Circular Economy Action Plan was implemented to help firms become more sustainable. In this sense, Govindan and Hasanagic (2018) demonstrated in an empirical study of the supply chain that the government is the actor that has the most impact on the implementation of the Circular Economy. In this context, it is surprising that the principal difficulty that firms identify is the complexity of administrative or legal requirements, except for Cluster 1, which focuses on the costs of these actions. Additionally, more environmentally proactive firms face the most difficulties. Further, after subsidies, firms’ second strongest call is for better cooperation between firms in Cluster 3 and for consultancy in Cluster 2. It seems that less proactive firms need technical assistance in implementing environmental actions.

In sum, first, most firms seem to find the implementation of environmentally sustainable practices to be expensive and entail administrative difficulties. As such, it is noteworthy that macro-level initiatives such as the Circular Economy Action Plan are considered a problem for firms rather than a source of help. Second, firms are seeking a more dynamic connection with their commercial counterparts and are requesting support to encourage meso-level initiatives. In this regard, the problem of asymmetric information could be best addressed by active engagement on the part of public administrations or bodies designed for this purpose. Finally, the least proactive cluster does not see a way forward to implement green practices.

All of these are considerations for policymakers to consider for the improvement of the sustainable economy process across the European Union.

## 5. Conclusions

This study grouped the European firms according to their environmental actions. We found three types of clusters, each with different characteristics and identified the countries in which each group was principally located.

The first cluster comprises the largest number of firms, mainly those that provide services to other firms. They are characterized by a strong focus on energy savings and waste minimization.

The second group, Cluster 2, stands out because it constitutes the firms that carry out the fewest actions.

At the other extreme, we have Cluster 3, whose main characteristic is that it carries out the most actions. It is also the most diverse cluster in terms of the type of targeted customer. Companies ask for financial support to become more environmentally sustainable, as they state that the actions carried out are costly and for more information, as they see a benefit in collaboration with other firms in this area. Difficulties in administrative processes are the second problem faced by firms, and firms also ask for help in implementing new technologies or processes to become more efficient.

In short, they request financial support, on the one hand, and, on the other hand, greater simplicity in terms of administrative procedures, along with information regarding possible cooperation between firms regarding new technologies or processes.

## Ethics and consent

Ethical approval and consent were not required for this study.

## Data Availability

All the data underlying the results presented in this article are publicly available under open licences permitting reuse. Garcia-Alcober, M. P., Mateos Ansótegui, A. I., Pastor Gosálbez, M. T., & Navarro, J. (2026). Extended data “How are European Small Enterprises dealing with the Circular Economy?” [Data set]. Zenodo. https://doi.org/10.5281/zenodo.19691797 García-Alcober (2026). Data are available under the terms of the Creative Commons Attribution 4.0 International. All datasets are freely available and distributed under open licenses permitting reuse (CC-BY or equivalent), depending on the source). These datasets can be used to replicate the analyses and findings presented in this study.

## Footnotes

^1^
A clustering mechanism for non-numeric data, K-modes, has been used. This procedure for grouping categorical data uses the simple coincidence similarity measure, using the modes to represent the cluster centres and updating the modes with the most frequent categorical values in each of the iterations of the clustering process. The dissimilarity metric used for K-modes is the information-theoretic Hamming distance. The mode vector minimizes the sum of the distances between each object in the cluster and the centre of the cluster. The analysis has been carried out using Python and the following libraries: Numpy, pandas, matplotlib, seaborn, kmodes (we used “cao” as an initializer). A priori it was assumed that the initial number of clusters was 3.

## References

[ref1] Baldassarre B : Circular economy for resource security in the European Union (EU): Case study, research framework, and future directions. *Ecol. Econ.* 2025;227:108345. 10.1016/j.ecolecon.2024.108345

[ref2] Cao F Liang J Bai L : A New Initialization Method for Categorical Data Clustering. *Expert Syst. Appl.* 2009;36(7):10223–10228. 10.1016/j.eswa.2009.01.060

[ref4] Chatzistamoulou N Tyllianakis E : Commitment of European SMEs to resource efficiency actions to achieve sustainability transition. A feasible reality or an elusive goal?. *J. Environ. Manag.* 2022;321:115937. 10.1016/j.jenvman.2022.115937 35988398

[ref5] Christis M Athanassiadis A Vercalsteren A : Implementation at a city level of circular economy strategies and climate change mitigation–the case of Brussels. *Journal of Cleaner Production.* 2019;218:511–520. 10.1016/j.jclepro.2019.01.180

[ref6] European Commission: *Closing the loop—An EU action plan for the circular economy.* Communication from the Commission to the European Parliament, the Council, the European Economic and Social Committee and the Committee of the Regions;2015.

[ref7] European Commission: A new Circular Economy Action Plan for a cleaner and more competitive Europe. 2020. Reference Source

[ref8] European Commission: *Flash Eurobarometer 498. SMEs, Resource Efficiency and Green Markets, wave 5.* GESIS; Cologne:2022a. ZA7806 Data file Version 1.0, Brussels 2022. 10.4232/1.13934

[ref9] European Commission: *Annual Report on European SMEs 2021/22. SMEs and environmental sustainability.* Brussels:2022b; vol.2022.

[ref10] European Parliament and Council: Regulation (EU) 2024/1252 of the European Parliament and of the Council of 11 April 2024 Establishing a Framework for Ensuring a Secure and Sustainable Supply of Critical Raw Materials and Amending Regulations (EU) no 168/2013, (EU) 2018/858, (EU) 2018/17. *Off. J. Eur. Union.* 2024;2024:1–67.

[ref21] Garcés-Ayerbe C Rivera-Torres P Suárez-Perales I : Is it possible to change from a linear to a circular economy? An overview of opportunities and barriers for European small and medium-sized enterprise companies. *Int. J. Environ. Res. Public Health.* 2019;16(5):851. 30857193 10.3390/ijerph16050851 PMC6427659

[ref12] García-Alcober M : Online Appendix to "How are European Small Enterprises dealing with the Circular Economy?". *Zenodo.* 2026. 10.5281/zenodo.19591312

[ref13] Ghisellini P Cialani C Ulgiati S : A review on circular economy: the expected transition to a balanced interplay of environmental and economic systems. *Journal of Cleaner Production.* 2016;114:11–32. 10.1016/j.jclepro.2015.09.007

[ref14] Gomonov K Ratner S Lazanyuk I : Clustering of EU countries by the level of circular economy: An objective-oriented approach. *Sustainability.* 2021;13:7158. 10.3390/su13137158

[ref15] Govindan K Hasanagic M : A systematic review on drivers, barriers, and practices towards circular economy: a supply chain perspective. *Int. J. Prod. Res.* 2018;56(1–2):278–311. 10.1080/00207543.2017.1402141

[ref22] Kirchherr J Piscicelli L Bour R : Barriers to the circular economy: evidence from the European Union (EU). *Ecological Economics.* 2018;150:264–272. 10.1016/j.ecolecon.2018.04.028

[ref17] Masi D Kumar V Garza-Reyes JA : Towards a more circular economy: exploring the awareness, practices, and barriers from a focal firm perspective. *Prod. Plan. Control.* 2018;29(6):539–550. 10.1080/09537287.2018.1449246

[ref23] Rizos V Behrens A Van der Gaast W : Implementation of Circular Economy Business Models by Small and Medium-Sized Enterprises (SMEs): Barriers and Enablers. *Sustainability.* 2016;8(11):1212. 10.3390/su8111212

[ref19] Turcu C Gillie H : Governing the circular economy in the city: Local planning practice in London. *Plan. Pract. Res.* 2020;35(1):62–85. 10.1080/02697459.2019.1703335

[ref20] Vasilescu MD Dimian GC Gradinaru GI : Green entrepreneurship in challenging times: a quantitative approach for European countries. *Economic research-Ekonomska istraživanja.* 2023;36(1):1828–1847. 10.1080/1331677X.2022.2093767

