# Supplementary material for: How are European small enterprises dealing with the circular economy?
Source: Open Res Eur. 2026 Oct 6;6:163. Originally published 2026 May 13. [Version 2] doi: 10.12688/openreseurope.23303.2 (PMC13650320; doi:10.12688/openreseurope.23303.2)
Supplement: Peer review history

# Reviewer report: Reviewer response for version 2

**DOI:** 10.21956/openreseurope.27144.r82634

**Published:** 2026-10-08

**Authors:** Grigorios L. Kyriakopoulos

**Affiliations:** 1 National Technical University of Athens, Athens, Greece

This revised manuscript can be accepted for indexing in the Open Research Europe journal as is.

If applicable, is the statistical analysis and its interpretation appropriate?

Partly

Is the study design appropriate and is the work technically sound?

Yes

Is the work clearly and accurately presented and does it engage with the current literature?

Yes

Are the conclusions drawn adequately supported by the results?

Yes

Are sufficient details of methods and analysis provided to allow replication by others?

Partly

Are all the source data and materials underlying the results available?

Yes

Reviewer Expertise:

Engineering, Environment, Renewable Energy, Analytical Techniques, Business and Management, Low Carbon Economy, Development Economics, Circular Economy, Behavioural Ecology

I confirm that I have read this submission and believe that I have an appropriate level of expertise to confirm that it is of an acceptable scientific standard.

## References

[1] García-Alcober MP, Mateos-Ansótegui AI, Pastor-Gosálbez MT and Navarro-Ortiz J. How are European small enterprises dealing with the circular economy?.

# Reviewer report: Reviewer response for version 1

**DOI:** 10.21956/openreseurope.25210.r76121

**Published:** 2026-07-14

**Authors:** Francisco Villegas Pinuer

**Affiliations:** 1 Universidad Diego Portales, Santiago, Chile

Dear authors,

 The article is interesting for its approximation of circular economy actions in the Eurobarometer data. 

 In this regard, there are some aspects to consider.

 Introduction

 This section should be reinforced in light of the circular economy hierarchy framework. The circular economy practices considered in the survey have varying levels of circularity, which lead to different environmental effects. In addition, the first paragraph would benefit from adding the circular economy definition of Geissdoerfer, considering their whole scope (materials, energy)

 Methodology

 The section is well presented. Nonetheless, 2.2 should be titled "Method" rather than "Methodology". Then, the procedure used should be explained in more detail, considering the underlying assumptions of the process.

 In addition, the presentation of grouped circular economy practices should be introduced in the first section of the article. In this regard, it is relevant to define the conceptual approximation of the circular economy phenomenon clearly.

 Results

 The section is straightforward in its presentation. The clustering description is concise. 

 Discussion

 The section needs to emphasize the article's contributions. Actually, the presentation is descriptive.

If applicable, is the statistical analysis and its interpretation appropriate?

Yes

Is the study design appropriate and is the work technically sound?

Yes

Is the work clearly and accurately presented and does it engage with the current literature?

Partly

Are the conclusions drawn adequately supported by the results?

Partly

Are sufficient details of methods and analysis provided to allow replication by others?

Partly

Are all the source data and materials underlying the results available?

Yes

Reviewer Expertise:

Environmental sustainability and circular economy

I confirm that I have read this submission and believe that I have an appropriate level of expertise to confirm that it is of an acceptable scientific standard, however I have significant reservations, as outlined above.

# Response

**Published:** 2026-08-27

**Authors:** Josep Navarro-Ortiz

**Affiliations:** Anàlisi Econòmic, Universitat de Valencia, Valencia, Valencian Community, Spain

**Dear authors,** **The article is interesting for its approximation of circular economy actions in the Eurobarometer data.** **In this regard, there are some aspects to consider.** **Introduction** **This section should be reinforced in light of the circular economy hierarchy framework. The circular economy practices considered in the survey have varying levels of circularity, which lead to different environmental effects. In addition, the first paragraph would benefit from adding the circular economy definition of Geissdoerfer, considering their whole scope (materials, energy)**

 Thank you for your comment. We have added a second part in the 1st paragraph mentioning Geissdoerfer et al. (2017) .We have also added a final paragraph noting that not all environmental activities are equally effective in achieving a circular economy. 

**Methodology** **The section is well presented. Nonetheless, 2.2 should be titled "Method" rather than "Methodology". Then, the procedure used should be explained in more detail, considering the underlying assumptions of the process.**

 Thank you for your observation. We have modified the section 2 “Methodology. We added a new subsection 2.1. “database”  to explain the data; We modified the name of the subsection 2.2. that now is “Analysis of the business structure in Europe. As a result, the “methodology” subsection has been renumbered as subsection 2.3. and is now titled “method.” 

**In addition, the presentation of grouped circular economy practices should be introduced in the first section of the article. In this regard, it is relevant to define the conceptual approximation of the circular economy phenomenon clearly.**

 We have added a new section 2.1. to explain the data. We have also categorized the actions taken based on their characteristics for better clarification. 

**Results** **The section is straightforward in its presentation. The clustering description is concise.** **Discussion** **The section needs to emphasize the article's contributions. Actually, the presentation is descriptive.**

 Thank you for your comment. We have rewritten the discussion based on your comments.

# Reviewer report: Reviewer response for version 1

**DOI:** 10.21956/openreseurope.25210.r76120

**Published:** 2026-06-30

**Authors:** Grigorios L. Kyriakopoulos

**Affiliations:** 1 National Technical University of Athens, Athens, Greece

A general comment is that authors include a number of different terms to mention the same thing, which are the terms of: enterprises, firms, companies, in different parts of their study. The consistent terminology throughout the text content, including Tables and Figures, should be mostly welcome.

 In section 1. Introduction, there should be a more solidified cross-citing or EU SMEs with the SDGs and the EU regulations, since this is a core emerging topic of theoretical coverage in the study.

 From data of Table 1, all numbers of companies per country to be summed and the total to be calculated. Then the percentages of each one participant country to be added in parentheses next to these numbers, for example in the case of Austria:  509 (…...percentage, to be added here…...%), and the same to the other countries.

 In subsection 2.1 authors mentioned that “We selected companies from 27 European Union member states. After the database was cleaned, 14,008 enterprises were included in the sample”. However, while this statement is clearly noted in Table 6, it is not clearly conveyed or outlined in data of Tables 1-5 and Figure 1, in which a reader should obtain the false perception/imply that the clustering and numerical data per country are representative for the whole country, but they are actually survey-derived allocations. This misconception is clearer in Table 4 in which the referencing of 1840 firms are contradicting to the sampling size of 14008 enterprises. Therefore, for these types of information, Tables 1-5 and Figure 1, it could be more realistic if -besides to survey-based allocation- authors could retrieve national-based, placing them either as separate Table-columns, or in parentheses next to the survey-based data.

 In Tables in which there is an extensive repetition of the percentage symbol (%) to all Table-columns, this percentage symbol (%) can be transferred in the corresponding Table-heading-line and to remove from the cells underneath it.

 In the y-axis of Figure 1, the missing legend and the percentage symbol (%) can be added, as: “Percentage of businesses (%)”. Besides, in Figures 3,4,5 the subgraphs have to be numbered as a, b, c, not 1,2,3, since this Arabic numbering 1,2, 3……, follows the main Figures, not their subfigures, for which the alphabetical numbering is suitable.

 In subsection 3.3 the term of “difficulties” sounds informal for scientific research. In my opinion the upgrading of the “difficulties” term can be approached by a more systematic and in-depth manner, thus, the adoption and presentation of the following 2 types of analysis: SWOT and PESTLE, can offer a more systematic approach of the findings. The formulation of relevant 2X2 Table, per dimension: Strengths, Weakness, Opportunities, Threads, can plausibly transforms the Difficulties (implied by the dimensions, W and T) into advantages S and O, respectively. Similarly, the dimensions of Political, Economic, Social, Technological, Legal, and Environmental can provide a more comprehensive understanding of the clustered companies in alignment with their perceptions of the difficulties to cope with when implementing environmental policy measures under intra-governmental EU restrictions and specifications, motivations, national rules and directions.

 In response of setting/study statements:

 -two-fold research objectives: “on the one hand, to analyze the responses of companies by country to the environmental measures proposed in the EU regulation, and on the other hand, to categorize companies in terms of the actions taken in response to these environmental measures on a country-by-country basis”

 -The research focuses on “a better understanding of SMEs in the context of a Circular Economy in the EU”,

 authors are recommended to elaborate the rather weakly developed section 4. Discussion by enriching it and succinctly addressing the following SMEs-oriented issues (indicatively) of environmental and circular economy interest among the conducted survey on the participating companies:

 -The role of extroversion and internationalization of the national markets towards a liberal international marketplace.

 -The contribution of microfinancing and environmental education in order to instill the pro-environmental perceptions in the SMEs trading.

 -The involvement of the digitalization and transaction economy types, which can activate the role of social communities and vulnerable social groups: women, children, migrants, in collecting recyclable materials and running FabLabs networks of digital fabrication laboratories through platforms for cooperation in urban locations.

 For this reason, the Discussion section can be extended up to one extra, and cross-citing, text page.

If applicable, is the statistical analysis and its interpretation appropriate?

Partly

Is the study design appropriate and is the work technically sound?

Yes

Is the work clearly and accurately presented and does it engage with the current literature?

Yes

Are the conclusions drawn adequately supported by the results?

Yes

Are sufficient details of methods and analysis provided to allow replication by others?

Partly

Are all the source data and materials underlying the results available?

Yes

Reviewer Expertise:

Engineering, Environment, Renewable Energy, Analytical Techniques, Business and Management, Low Carbon Economy, Development Economics, Circular Economy, Behavioural Ecology

I confirm that I have read this submission and believe that I have an appropriate level of expertise to confirm that it is of an acceptable scientific standard, however I have significant reservations, as outlined above.

# Response

**Published:** 2026-08-27

**Authors:** Josep Navarro-Ortiz

**Affiliations:** Anàlisi Econòmic, Universitat de Valencia, Valencia, Valencian Community, Spain

**A general comment is that authors include a number of different terms to mention the same thing, which are the terms of: enterprises, firms, companies, in different parts of their study. The consistent terminology throughout the text content, including Tables and Figures, should be mostly welcome.**

 Thank you for your observation. We have modified, we use the term “firms” in the text 

**In section 1. Introduction, there should be a more solidified cross-citing or EU SMEs with the SDGs and the EU regulations, since this is a core emerging topic of theoretical coverage in the study.**

 Thank you for your comment. We have replaced the third paragraph with a new one, reinforcing the proposed idea. 

**From data of Table 1, all numbers of companies per country to be summed and the total to be calculated. Then the percentages of each one participant country to be added in parentheses next to these numbers, for example in the case of Austria:  509 (…...percentage, to be added here…...%), and the same to the other countries.**

 Thank you for your observation. We have modified. We considered the data provided by the survey; it did not include the total number of companies per country. We have rewritten the text for a better understanding. 

**In subsection 2.1 authors mentioned that “We selected companies from 27 European Union member states. After the database was cleaned, 14,008 enterprises were included in the sample”. However, while this statement is clearly noted in Table 6, it is not clearly conveyed or outlined in data of Tables 1-5 and Figure 1, in which a reader should obtain the false perception/imply that the clustering and numerical data per country are representative for the whole country, but they are actually survey-derived allocations. This misconception is clearer in Table 4 in which the referencing of 1840 firms are contradicting to the sampling size of 14008 enterprises. Therefore, for these types of information, Tables 1-5 and Figure 1, it could be more realistic if -besides to survey-based allocation- authors could retrieve national-based, placing them either as separate Table-columns, or in parentheses next to the survey-based data. ** Thank you for your comment. The study analyses the Eurobarometer survey sample rather than the complete population. Table 4 represents only firms over 50 million €, not the total of firms. We rewrite the 1st paragraph of this section to ensure that it´s clear. 

**In Tables in which there is an extensive repetition of the percentage symbol (%) to all Table-columns, this percentage symbol (%) can be transferred in the corresponding Table-heading-line and to remove from the cells underneath it**

 Thank you for your comment. We removed the percentage symbol from the cells. 

**In the y-axis of Figure 1, the missing legend and the percentage symbol (%) can be added, as: “Percentage of businesses (%)”. Besides, in Figures 3,4,5 the subgraphs have to be numbered as a, b, c, not 1,2,3, since this Arabic numbering 1,2, 3……, follows the main Figures, not their subfigures, for which the alphabetical numbering is suitable.**

 Thank you for your comment. We added the legend and modified the subgraphs numbering. 

**In subsection 3.3 the term of “difficulties” sounds informal for scientific research. In my opinion the upgrading of the “difficulties” term can be approached by a more systematic and in-depth manner, thus, the adoption and presentation of the following 2 types of analysis: SWOT and PESTLE, can offer a more systematic approach of the findings. The formulation of relevant 2X2 Table, per dimension: Strengths, Weakness, Opportunities, Threads, can plausibly transforms the Difficulties (implied by the dimensions, W and T) into advantages S and O, respectively. Similarly, the dimensions of Political, Economic, Social, Technological, Legal, and Environmental can provide a more comprehensive understanding of the clustered companies in alignment with their perceptions of the difficulties to cope with when implementing environmental policy measures under intra-governmental EU restrictions and specifications, motivations, national rules and directions.**

 Thank you for your comment. We modified the term “difficulties” by “barriers”. About the SWOT and PESTLE analysis, it´s out of our focus. A SWOT and PESTLE analysis could be use on a new paper using new databases and different information for all the different countries. Your contribution is very interesting and we´ll estimate on another paper.

**In response of setting/study statements:** **-two-fold research objectives: “on the one hand, to analyze the responses of companies by country to the environmental measures proposed in the EU regulation, and on the other hand, to categorize companies in terms of the actions taken in response to these environmental measures on a country-by-country basis”** **-The research focuses on “a better understanding of SMEs in the context of a Circular Economy in the EU”,** **authors are recommended to elaborate the rather weakly developed section 4. Discussion by enriching it and succinctly addressing the following SMEs-oriented issues (indicatively) of environmental and circular economy interest among the conducted survey on the participating companies:** **-The role of extroversion and internationalization of the national markets towards a liberal international marketplace.** **-The contribution of microfinancing and environmental education in order to instill the pro-environmental perceptions in the SMEs trading.** **-The involvement of the digitalization and transaction economy types, which can activate the role of social communities and vulnerable social groups: women, children, migrants, in collecting recyclable materials and running FabLabs networks of digital fabrication laboratories through platforms for cooperation in urban locations.** **For this reason, the Discussion section can be extended up to one extra, and cross-citing, text page.**

 Thank you for your asportation. We improved the discussion section. We include new references and comments. Nevertheless, we are using a Eurobarometer database that doesn´t contain so many indicators. The analysis proposed is very interesting, however, it´s not our focus and we don´t have so much information.

# Reviewer report: Reviewer response for version 1

**DOI:** 10.21956/openreseurope.25210.r75787

**Published:** 2026-06-27

**Authors:** Antonis Skouloudis

**Affiliations:** 1 University of the Aegean, Lesvos, Greece

The study is a useful descriptive baseline, but in its current form several choices and inconsistencies reduce confidence in the precision of the findings. The most important is that the segmentation is partly circular: the variables used to test cluster differences are the same variables used to construct the clusters.

 Alongside this, the number of clusters is fixed a priori without selection diagnostics, the role of survey weights in the clustering and inference is unclear, and there are reproducible errors in several tables. With methodological clarification, sensitivity analysis and correction of the descriptive inconsistencies, the paper would meet the expected standards.

 Major points:

- The nine environmental actions are used as inputs to the K-modes clustering, and Table 10 then tests the association between those same nine actions and the resulting clusters using chi-square and Cramér's V. Variables used to define the clusters will, almost by construction, show strong associations with them, so the very large statistics reported (chi-square up to 4,463; Cramér's V up to 0.564) are largely tautological and do not constitute independent validation of the segmentation. I recommend either (i) clustering on the environmental actions only and then testing genuinely independent variables (sector, size, country, output type) against cluster membership, or (ii) presenting the action-cluster associations explicitly as a description of how the algorithm partitioned the data rather than as evidence of significance. The same caution applies to Table 11 if those variables also entered the clustering.
- Number of clusters fixed a priori without selection evidence: the footnote on p.7 states that the initial number of clusters was assumed to be three. No model-selection procedure is reported (e.g. a cost or elbow curve for K-modes, stability across random seeds, or an internal validity index suitable for categorical data). Please report diagnostics across a range of k (for example k = 2 to 6) and justify the choice of three on both fit and interpretability grounds.
- Inclusion of “country” as a clustering feature: country is listed on p.7 among the variables fed into K-modes, yet the analysis subsequently compares clusters across countries (fig.6, table 12). Using geography to build the clusters and then interpreting their geographic distribution risks confounding behaviour with location. I suggest clustering on the behavioural variables only and profiling clusters by country, sector and size ex post, with a sensitivity run that includes the attributes so readers can see how robust the partition is.
- Treatment of survey weights in clustering and inference is unclear. The descriptive section (page 4) notes that the data were weighted to the SME population in each country, but it is not stated whether weights were applied in the clustering or in the chi-square and Cramér's V calculations. This matters in two ways: unweighted clustering may bias the segments given cross-country differences in sample composition, and chi-square statistics computed on weighted counts are not interpretable in the usual way because the effective sample size is distorted. Please state explicitly how weights were handled and, ideally, compare weighted and unweighted results.

- The paper frames the analysis around SMEs throughout, yet discusses 164 firms with turnover above €50 million, which exceeds the EU SME turnover ceiling. Please either justify their inclusion or report whether the findings are materially affected when these firms are excluded or when results are stratified by size class (micro, small, medium).

Further points for improvement:

- Accuracy of the headline emissions figure. The abstract and introduction state that European SMEs account for 63.3% of total CO₂ emissions in Europe. The cited source (European Commission, 2022, Annual Report on European SMEs 2021/22) reports 63.3% of CO₂ and greenhouse-gas emissions by enterprises, that is, within the business sector, which excludes households, agriculture and much of transport. “Total CO₂ emissions” overstates the figure and should be corrected to refer to emissions by enterprises or the business sector.
- Non-standard description of the dissimilarity metric. Footnote 1 (p.7) is internally inconsistent: it describes both a “simple coincidence similarity measure” (which is correct for K-modes) and an “information-theoretic Hamming distance” (which is not standard terminology and not the default of the kmodes package; “cao” refers to the initialisation method, not the dissimilarity). Please correct the terminology and confirm the exact objective function used.
- Transposed captions on Tables 4 and 5. The caption of Table 4 reads “turnover of over €50million,” but its contents (Greece, Latvia, Malta, Romania, Slovakia, with shares of 71 - 81%) and its column header describe the sub-2M group. Conversely, Table 5 is captioned “turnover below 2 million euros” but its contents (Belgium, Denmark, Sweden; total 164 firms, 38%) describe the above-€ 50 million group. The captions appear to be swapped and should be corrected.
- Table 13 errors: wo pairs of rows are identical across all three clusters and the chi-square value (“lack of demand for resource-efficient products” and “complexity associated with environmental labelling” are both 17.27 / 8.72 / 26.48, chi-square 2,496.42; “lack of specific environmental expertise” and “cost of environmental actions” are both 24.08 / 13.54 / 41.33, chi-square 2,715.98). Duplicated rows suggest a copy-paste error. In addition, the latter two rows share an identical chi-square (2,715.98) but are assigned different Cramér's V values (0.424 and 0.422), which is not possible at equal degrees of freedom and sample size. Please re-check the underlying numbers.
- Inconsistent listing of the six firm characteristics; in p.7 lists the characterising variables as country, productive sector, size, level of growth, type of product and type of customer, whereas Table 11 lists country, output, “to whom do you sell,” productive sector, size and revenues from last year. “Level of growth” and “revenues from last year” are not obviously the same variable. Please reconcile the two descriptions and state precisely which variables entered the algorithm and which were used only for profiling.
- On p.14 the authors cite Table 6 to support the contrast between Swedish (design-oriented) and Spanish (cost-oriented) behaviour, but Table 6 reports output type (products, services or both). The intended reference appears to be Table 7, or figs 3 to 5.
- Area coding (A to Z) underspecified; the mapping in Figure 6 and Table 12 uses areas A, B, C and the mixed categories X, Y and Z. The threshold that defines a “predominant” cluster, and the rule used to assign the mixed cases, are not stated. Please quantify these so the map can be reproducible.
- The text notes only that the database was “cleaned” to 14,008 firms. Please report how missing or “don’t know” responses across the categorical variables were handled, whether any categories were collapsed, and how this might affect cluster composition.
- The underlying data are openly archived (Zenodo). Given the table errors noted above, sharing the analysis code (the -python- scripts used for clustering and the contingency analyses) would allow numerical inconsistencies to be resolved transparently.
- A careful proofreading is needed - e.g. check the sentence on p.9 (“72.19 Of the Spanish companies, 72.19%”), “elaboration of our own,” the sentence in the Data availability statement, the Spanish-derived action codes in Table 7 (AA, AE, AM and so on) used before they’re defined on p.9, “Tyllianankis” vs. “Tyllianakis” in the ref list.

The manuscript treats the barriers-and-enablers literature thinly relative to its empirical focus. The discussion of why administrative complexity and cost dominate, and why cooperation and consultancy are sought, would benefit from sustained engagement with the established empirical work on SME circular-economy barriers and drivers. I suggest the authors draw on, among others:

  

- Rizos et al. (2016), on barriers - enablers to circular-economy business models in SMEs
- Kirchherr et al. (2018), on barriers to the circular economy with EU evidence
- Garcés-Ayerbe et al. (2019), on opportunities-barriers for EU SMEs in the linear-to-circular transition

These could/would help the authors position their descriptive findings against prior cross-national evidence and sharpen the discussion of the policy levers they identify.

 The following suggestion is optional and is not a condition of approval. I mention the following papers (whose I’m a co-author) only because they bear directly on the enablers, barriers and performance dimensions that this paper raises and draw on European, and specifically Spanish and Greek, SME samples that overlap with the cases highlighted here. Should the authors find them useful for the literature review or the discussion of barriers and support needs, they may wish to consult Moursellas et al. (2023) on sustainability practices and performance in European SMEs, Moursellas et al. (2024) on perceived enablers and barriers across four European countries, and Moursellas et al. (2026) on sustainability dynamics in Greek SMEs.

 <><><>

 European Commission (2022) Annual Report on European SMEs 2021/22: SMEs and environmental sustainability. Luxembourg: Publications Office of the European Union.

 Garcés-Ayerbe, C., Rivera-Torres, P., Suárez-Perales, I. and Leyva-de la Hiz, D.I. (2019) ‘Is it possible to change from a linear to a circular economy? An overview of opportunities and barriers for European Union SMEs’, International Journal of Environmental Research and Public Health, 16(5), 851.

 Kirchherr, J., Piscicelli, L., Bour, R., Kostense-Smit, E., Muller, J., Huibrechtse-Truijens, A. and Hekkert, M. (2018) ‘Barriers to the circular economy: evidence from the European Union (EU)’, Ecological Economics, 150, pp. 264–272.

 Moursellas, A., De, D., Wurzer, T., Skouloudis, A., Reiner, G., Chaudhuri, A., Manousidis, T., Malesios, C., Evangelinos, K. and Dey, P.K. (2023) ‘Sustainability practices and performance in European small-and-medium enterprises: insights from multiple case studies’, Circular Economy and Sustainability, 3(2), pp. 835–860.

 Moursellas, A., Malesios, C., Skouloudis, A., Evangelinos, K. and Dey, P.K. (2024) ‘Perceived enablers and barriers impacting sustainability of small-and-medium sized enterprises: a quantitative analysis in four European countries’, Environmental Quality Management, 33(3), pp. 433–448.

 Moursellas, A., Malesios, C., Skouloudis, A., Kantartzis, A., Sepetis, A., Sfakianaki, E. and Evangelinos, K. (2026) ‘Sustainability dynamics in Greek small and medium-sized enterprises: a multimethod analysis’, Journal of the International Council for Small Business (advance online publication).

 Rizos, V., Behrens, A., van der Gaast, W., Hofman, E., Ioannou, A., Kafyeke, T., Flamos, A., Rinaldi, R., Papadelis, S., Hirschnitz-Garbers, M. and Topi, C. (2016) ‘Implementation of circular economy business models by small and medium-sized enterprises (SMEs): barriers and enablers’, Sustainability, 8(11), 1212.

If applicable, is the statistical analysis and its interpretation appropriate?

Partly

Is the study design appropriate and is the work technically sound?

Partly

Is the work clearly and accurately presented and does it engage with the current literature?

Partly

Are the conclusions drawn adequately supported by the results?

Partly

Are sufficient details of methods and analysis provided to allow replication by others?

Not applicable

Are all the source data and materials underlying the results available?

Yes

Reviewer Expertise:

organisational sustainability; SMEs; nonfinancial performance

I confirm that I have read this submission and believe that I have an appropriate level of expertise to confirm that it is of an acceptable scientific standard, however I have significant reservations, as outlined above.

# Response

**Published:** 2026-08-27

**Authors:** Josep Navarro-Ortiz

**Affiliations:** Anàlisi Econòmic, Universitat de Valencia, Valencia, Valencian Community, Spain

**The study is a useful descriptive baseline, but in its current form several choices and inconsistencies reduce confidence in the precision of the findings. The most important is that the segmentation is partly circular: the variables used to test cluster differences are the same variables used to construct the clusters.** **Alongside this, the number of clusters is fixed a priori without selection diagnostics, the role of survey weights in the clustering and inference is unclear, and there are reproducible errors in several tables. With methodological clarification, sensitivity analysis and correction of the descriptive inconsistencies, the paper would meet the expected standards.** **Major points:** **The nine environmental actions are used as inputs to the K-modes clustering, and Table 10 then tests the association between those same nine actions and the resulting clusters using chi-square and Cramér's V. Variables used to define the clusters will, almost by construction, show strong associations with them, so the very large statistics reported (chi-square up to 4,463; Cramér's V up to 0.564) are largely tautological and do not constitute independent validation of the segmentation.** **I recommend either  **

1. **clustering on the environmental actions only and then testing genuinely independent variables (sector, size, country, output type) against cluster membership,  **

1. **presenting the action-cluster associations explicitly as a description of how the algorithm partitioned the data rather than as evidence of significance. The same caution applies to Table 11 if those variables also entered the clustering. **

**Number of clusters fixed a priori without selection evidence: the footnote on p.7 states that the initial number of clusters was assumed to be three. No model-selection procedure is reported (e.g. a cost or elbow curve for K-modes, stability across random seeds, or an internal validity index suitable for categorical data). Please report diagnostics across a range of k (for example k = 2 to 6) and justify the choice of three on both fit and interpretability grounds.** **Inclusion of “country” as a clustering feature: country is listed on p.7 among the variables fed into K-modes, yet the analysis subsequently compares clusters across countries (fig.6, table 12). Using geography to build the clusters and then interpreting their geographic distribution risks confounding behaviour with location. I suggest clustering on the behavioural variables only and profiling clusters by country, sector and size ex post, with a sensitivity run that includes the attributes so readers can see how robust the partition is.**

 We thank the reviewer for this important observation. We agree that, since the nine environmental actions were included as input variables in the K-modes procedure, the chi-square tests and Cramér's V statistics reported in the previous version should not be interpreted as independent statistical validation of the resulting clusters. The strong associations partly reflect the fact that these variables contributed directly to the construction of the clusters.  Our analytical objective, however, differs from that of a clustering exercise based exclusively on environmental behaviour. The purpose of the study is to identify multidimensional firm profiles combining environmental practices with firms' structural and market characteristics. For this reason, environmental actions, country, sector, size, turnover evolution, type of output and customer orientation were intentionally incorporated into the clustering procedure. Removing the structural variables from K-modes would therefore change the analytical objective of the study rather than simply provide an alternative validation procedure.  Following the reviewer's recommendation, we have adopted option (ii). Specifically, we have removed the chi-square tests and Cramér's V statistics from the revised manuscript (tables 10 and 11) and no longer interpret the associations between the clustering variables and cluster membership as evidence of statistical significance or independent validation. Instead, these results are presented descriptively to show how the K-modes algorithm partitioned the observations and to facilitate interpretation of the multidimensional profiles obtained.  We have also revised the interpretation of the association matrix accordingly. The matrix is retained only as a descriptive tool for examining the association structure among the variables within the resulting profiles and is not presented as an independent validation of the clustering solution.  About the number of clusters, we estimated it with a cost or elbow curve for K-modes. This analysis has been added in an annex at the end of the paper. 

**Treatment of survey weights in clustering and inference is unclear. The descriptive section (page 4) notes that the data were weighted to the SME population in each country, but it is not stated whether weights were applied in the clustering or in the chi-square and Cramér's V calculations. This matters in two ways: unweighted clustering may bias the segments given cross-country differences in sample composition, and chi-square statistics computed on weighted counts are not interpretable in the usual way because the effective sample size is distorted. Please state explicitly how weights were handled and, ideally, compare weighted and unweighted results.**

 We have rewritten the text to clarify. We didn´t weighted the SMEs population, we just used the Eurobarometer database. 

**The paper frames the analysis around SMEs throughout, yet discusses 164 firms with turnover above €50 million, which exceeds the EU SME turnover ceiling. Please either justify their inclusion or report whether the findings are materially affected when these firms are excluded or when results are stratified by size class (micro, small, medium). ** We used the data as proposed by the Eurobarometer. Although, the large firms represent only the 0,01% of the sample. We didn´t remove this firms in order to maintain the same sample proposed by the Eurobarometer. 

**Further points for improvement:** **Accuracy of the headline emissions figure. The abstract and introduction state that European SMEs account for 63.3% of total CO₂ emissions in Europe. The cited source (European Commission, 2022, Annual Report on European SMEs 2021/22) reports 63.3% of CO₂ and greenhouse-gas emissions by enterprises, that is, within the business sector, which excludes households, agriculture and much of transport. “Total CO₂ emissions” overstates the figure and should be corrected to refer to emissions by enterprises or the business sector. ** Thank you for the observation. We have specified in the introduction in the abstract and paragraph 7 that we are referring to firms only. 

**Non-standard description of the dissimilarity metric. Footnote 1 (p.7) is internally inconsistent: it describes both a “simple coincidence similarity measure” (which is correct for K-modes) and an “information-theoretic Hamming distance” (which is not standard terminology and not the default of the kmodes package; “cao” refers to the initialisation method, not the dissimilarity). Please correct the terminology and confirm the exact objective function used.**

 Thank you for your comment. We modified the structure of the Method section. We added a new section 2.1 with database descriptions, and we have rewritten section 2.2. for a better understanding. Moreover, we provided more specifications of the method used in the annex. 

**Transposed captions on Tables 4 and 5. The caption of Table 4 reads “turnover of over €50million,” but its contents (Greece, Latvia, Malta, Romania, Slovakia, with shares of 71 - 81%) and its column header describe the sub-2M group. Conversely, Table 5 is captioned “turnover below 2 million euros” but its contents (Belgium, Denmark, Sweden; total 164 firms, 38%) describe the above-€ 50 million group. The captions appear to be swapped and should be corrected.**

 Thank you for the observation. There was an error and we modified tables 4 and 5. 

**Table 13 errors: wo pairs of rows are identical across all three clusters and the chi-square value (“lack of demand for resource-efficient products” and “complexity associated with environmental labelling” are both 17.27 / 8.72 / 26.48, chi-square 2,496.42; “lack of specific environmental expertise” and “cost of environmental actions” are both 24.08 / 13.54 / 41.33, chi-square 2,715.98). Duplicated rows suggest a copy-paste error. In addition, the latter two rows share an identical chi-square (2,715.98) but are assigned different Cramér's V values (0.424 and 0.422), which is not possible at equal degrees of freedom and sample size. Please re-check the underlying numbers. HECHO, revisar en 2024 10 ciencia regional la fila 197 y 199**

 Thank you for the observation. There was an error with different Cramér's V values (0.424 and 0.422), we modified it in the table.   

**Inconsistent listing of the six firm characteristics; in p.7 lists the characterising variables as country, productive sector, size, level of growth, type of product and type of customer, whereas Table 11 lists country, output, “to whom do you sell,” productive sector, size and revenues from last year. “Level of growth” and “revenues from last year” are not obviously the same variable. Please reconcile the two descriptions and state precisely which variables entered the algorithm and which were used only for profiling.**

 Thank you for your comment. Table 11 was confusing and did not provide any useful information. We have removed it. In addition, we have added Section 2.1, which specifies the variables to be analyzed. 

**On p.14 the authors cite Table 6 to support the contrast between Swedish (design-oriented) and Spanish (cost-oriented) behaviour, but Table 6 reports output type (products, services or both). The intended reference appears to be Table 7, or figs 3 to 5.**

 Thank you for your comment. We modified the text and substitute “Table 6” for “Table 7” 

**Area coding (A to Z) underspecified; the mapping in Figure 6 and Table 12 uses areas A, B, C and the mixed categories X, Y and Z. The threshold that defines a “predominant” cluster, and the rule used to assign the mixed cases, are not stated. Please quantify these so the map can be reproducible.**

 Thank you for your comment. We clarified it in the text, before figure 6 we introduced a new paragraph explaining what is considered a “predominant” cluster. 

**The text notes only that the database was “cleaned” to 14,008 firms. Please report how missing or “don’t know” responses across the categorical variables were handled, whether any categories were collapsed, and how this might affect cluster composition.**  Thank you for your comment. We added more specifications in the text paragraph 2.2. 

**The underlying data are openly archived (Zenodo). Given the table errors noted above, sharing the analysis code (the -python- scripts used for clustering and the contingency analyses) would allow numerical inconsistencies to be resolved transparently.**

 Thank you. We have shared the package python used in the section “method” part 2.2. More specifications in the annex. We removed the contingency analyses of table 10. The codes for tables 12 “perceive barriers contingency analyses” and 13 “request contingency analyses” have been included in the annex II. 

**A careful proofreading is needed - e.g. check the sentence on p.9 (“72.19 Of the Spanish companies, 72.19%”), “elaboration of our own,” the sentence in the Data availability statement, the Spanish-derived action codes in Table 7 (AA, AE, AM and so on) used before they’re defined on p.9, “Tyllianankis” vs. “Tyllianakis” in the ref list. ** Thank you. We modified the action codes according to English language (SW Saving Water, …). We also modified grammar errors for the text and bibliography. 

**The manuscript treats the barriers-and-enablers literature thinly relative to its empirical focus. The discussion of why administrative complexity and cost dominate, and why cooperation and consultancy are sought, would benefit from sustained engagement with the established empirical work on SME circular-economy barriers and drivers. I suggest the authors draw on, among others:** **Rizos et al. (2016), on barriers - enablers to circular-economy business models in SMEs** **Kirchherr et al. (2018), on barriers to the circular economy with EU evidence** **Garcés-Ayerbe et al. (2019), on opportunities-barriers for EU SMEs in the linear-to-circular transition** **These could/would help the authors position their descriptive findings against prior cross-national evidence and sharpen the discussion of the policy levers they identify.** **The following suggestion is optional and is not a condition of approval. I mention the following papers (whose I’m a co-author) only because they bear directly on the enablers, barriers and performance dimensions that this paper raises and draw on European, and specifically Spanish and Greek, SME samples that overlap with the cases highlighted here. Should the authors find them useful for the literature review or the discussion of barriers and support needs, they may wish to consult Moursellas et al. (2023) on sustainability practices and performance in European SMEs, Moursellas et al. (2024) on perceived enablers and barriers across four European countries, and Moursellas et al. (2026) on sustainability dynamics in Greek SMEs.** **<><><>** **European Commission (2022) Annual Report on European SMEs 2021/22: SMEs and environmental sustainability. Luxembourg: Publications Office of the European Union.** **Garcés-Ayerbe, C., Rivera-Torres, P., Suárez-Perales, I. and Leyva-de la Hiz, D.I. (2019) ‘Is it possible to change from a linear to a circular economy? An overview of opportunities and barriers for European Union SMEs’, International Journal of Environmental Research and Public Health, 16(5), 851.** **Kirchherr, J., Piscicelli, L., Bour, R., Kostense-Smit, E., Muller, J., Huibrechtse-Truijens, A. and Hekkert, M. (2018) ‘Barriers to the circular economy: evidence from the European Union (EU)’, Ecological Economics, 150, pp. 264–272.** **Moursellas, A., De, D., Wurzer, T., Skouloudis, A., Reiner, G., Chaudhuri, A., Manousidis, T., Malesios, C., Evangelinos, K. and Dey, P.K. (2023) ‘Sustainability practices and performance in European small-and-medium enterprises: insights from multiple case studies’, Circular Economy and Sustainability, 3(2), pp. 835–860.** **Moursellas, A., Malesios, C., Skouloudis, A., Evangelinos, K. and Dey, P.K. (2024) ‘Perceived enablers and barriers impacting sustainability of small-and-medium sized enterprises: a quantitative analysis in four European countries’, Environmental Quality Management, 33(3), pp. 433–448.** **Moursellas, A., Malesios, C., Skouloudis, A., Kantartzis, A., Sepetis, A., Sfakianaki, E. and Evangelinos, K. (2026) ‘Sustainability dynamics in Greek small and medium-sized enterprises: a multimethod analysis’, Journal of the International Council for Small Business (advance online publication).** **Rizos, V., Behrens, A., van der Gaast, W., Hofman, E., Ioannou, A., Kafyeke, T., Flamos, A., Rinaldi, R., Papadelis, S., Hirschnitz-Garbers, M. and Topi, C. (2016) ‘Implementation of circular economy business models by small and medium-sized enterprises (SMEs): barriers and enablers’, Sustainability, 8(11), 1212.**

 Thank you for your comments, we included new bibliography proposed among the paper: Garcés-Ayerbe et al., 2019; Kirchherr et al., 2018; Rizos et al., 2016; Mourellas et al., 2024.
